# Advances in Anion Chemistry in the Electrolyte Design for Better Lithium Batteries

**DOI:** 10.1007/s40820-024-01629-5

**Published:** 2025-02-17

**Authors:** Hecong Xiao, Xiang Li, Yongzhu Fu

**Affiliations:** College of Chemistry, Zhengzhou University, Zhengzhou, 450001 People’s Republic of China

**Keywords:** Anion chemistry, Electrolyte, Interface, Solvation structure

## Abstract

Electrolytes are crucial components in electrochemical energy storage devices, sparking considerable research interest. However, the significance of anions in the electrolytes is often underestimated. In fact, the anions have significant impacts on the performance and stability of lithium batteries. Therefore, comprehensively understanding anion chemistry in electrolytes is of crucial importance. Herein, in-depth comprehension of anion chemistry and its positive effects on the interface, solvation structure of Li-ions, as well as the electrochemical performance of the batteries have been emphasized and summarized. This review aims to present a full scope of anion chemistry and furnish systematic cognition for the rational design of advanced electrolytes for better lithium batteries with high energy density, lifespan, and safety. Furthermore, insightful analysis and perspectives based on the current research are proposed. We hope that this review sheds light on new perspectives on understanding anion chemistry in electrolytes.

## Highlights

- The impact of anions on the interface is summarized, including forming a solid electrolyte interphase (SEI), repairing the damaged SEI, and modulate electric double layer.
- The influence of anions on the solvation structure is presented, including enhancing desolvation process of the Li-ions and the antioxidant property of the electrolyte.
- This review also emphasizes the important role of anions in enhancing battery safety through their flame-retardant properties, as well as their impact on energy density and power density by altering reaction pathways and accelerating reactions.

## Graphical abstract

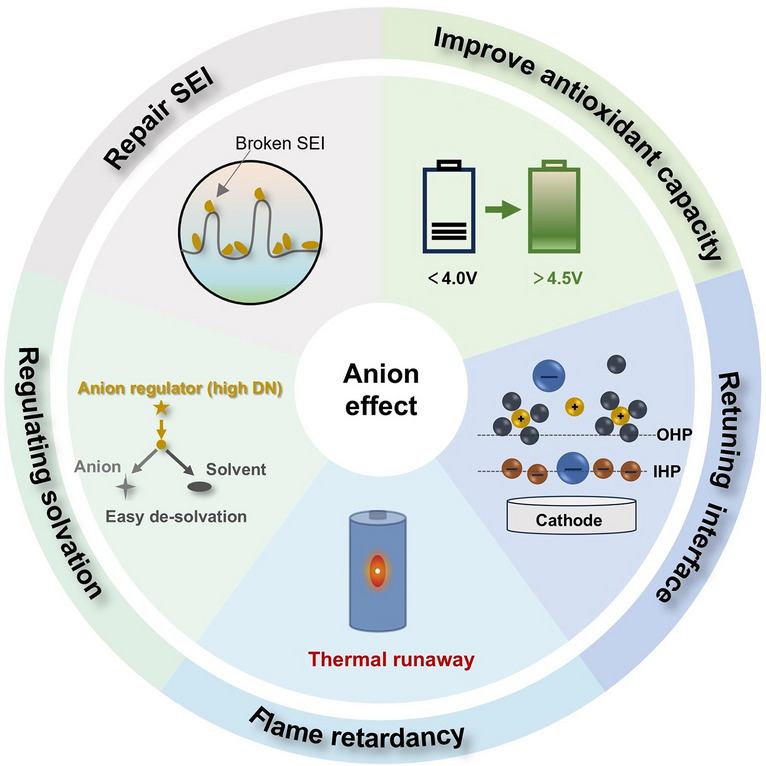

## Introduction

In today’s energy field, lithium-ion battery (LIB) is one important electrochemical energy storage device, which has closely connected with our daily life. With the increasing requirement of sustainability, durable LIBs with long lifespans and safety are highly needed [1, 2]. The electrolyte, as one of the core components of batteries, bears the important responsibility of ensuring the stability and performance of the battery during the operation [3–5]. It is well known that there are some typical electrolyte models, such as high-concentration electrolyte (HCE) [6–9], localized high-concentration electrolyte (LHCE) [10, 11], and weakly solvating electrolyte (WSE) [12–15], all of which focus on the solvents, such as decreasing the proportion of solvents and modifying the solvating ability of the solvents. Recently, the anions of Li-salts are applied for regulating the solvation structure of the electrolytes [16–18], which is a facile and effective strategy to achieve good electrochemical performance of LIBs. The anion is also an important component of the electrolyte, the effect of which, however, is often underestimated or overlooked.

Recent studies have shown that anions play multiple roles in interfacial electrochemistry and tuning the solvation sheath of Li-ions, which is very important for the formation of a solid electrolyte interphase (SEI) [19–23], the reaction kinetics at the electrode surface, and the stability of electrochemical performances [24–26]. Firstly, anions can decompose and help to form a solid SEI on the electrode surface [27–29], which is crucial for preventing solvents and salts from irreversible reactions at the electrode surface. Secondly, anions can participate in repairing the damaged SEI layer [30–32], thereby improving the stability of electrochemical performance and extending the electrode’s lifespan. Additionally, anions can regulate the structure of the electric double layer (EDL) between the electrolyte and electrode surface [32–34], affecting the kinetic of charge transfer, ion transport, and distribution of species at interfaces. By adjusting the solvation environment, anions can also enhance the electrolyte’s antioxidative and desolvation capabilities [35–38], thereby reducing side reactions in the electrolyte and improving battery cycle life. Furthermore, certain specific anions, such as halogens, possess flame-retardant properties, which can enhance battery safety [39, 40]. In recent years, Zhi et al. have thoroughly elucidated the critical role of anion chemistry in various energy storage devices, such as supercapacitors, cation rechargeable batteries, and metal–oxygen batteries [41]. Zhang et al. have emphasized the historical evolution and fundamental properties of salt anions [42]. The previous works provide profound insights into the critical role of anion chemistry in energy storage devices, while the design and functionalization of salt anions in new battery systems are not mentioned.

**Fig. 1:**
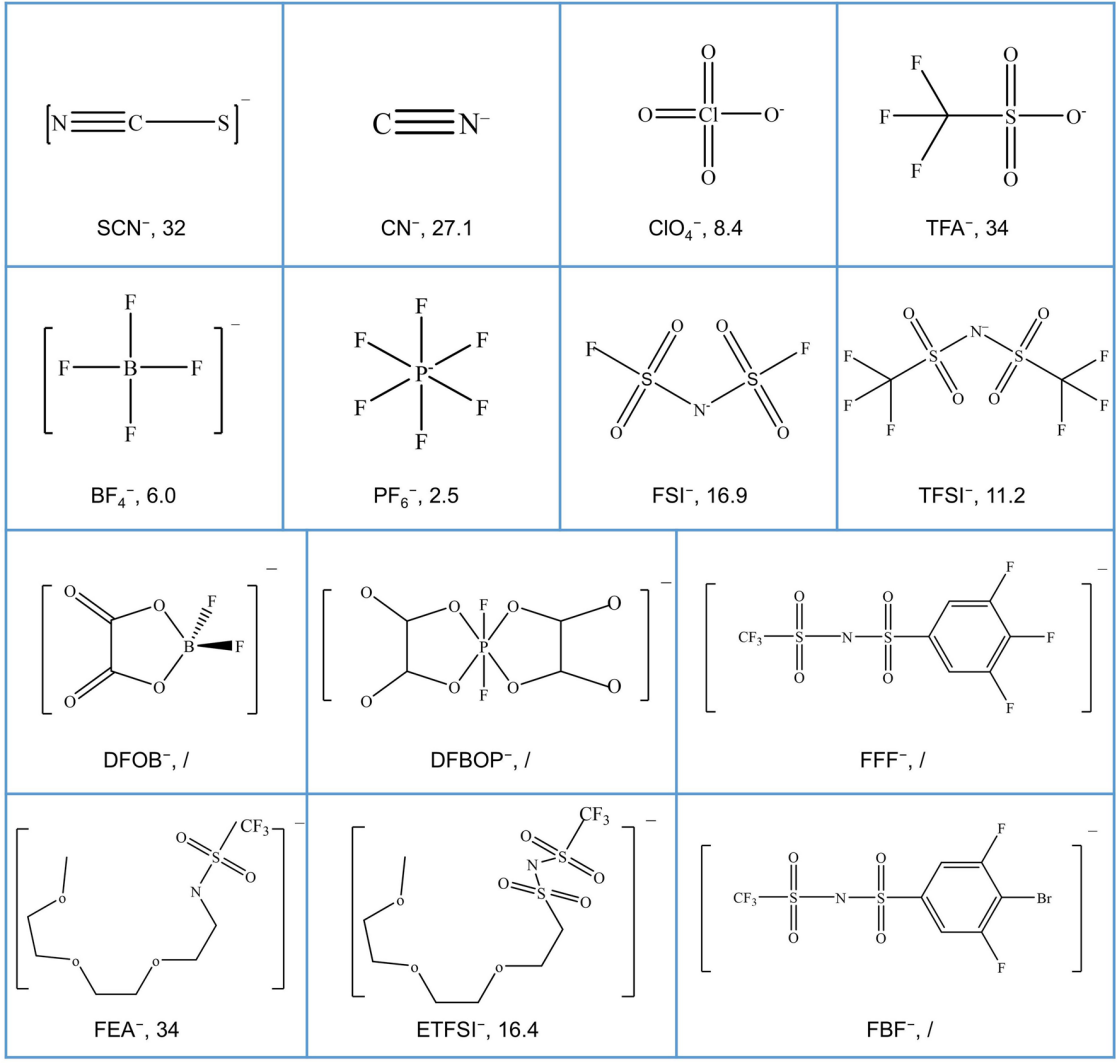
The structures of the typical anions involved in this review, together with the DN values

In this review, we will provide a detailed introduction to the key roles of anions, focusing on the precise design of anion structure and properties for better design of the LIBs (mainly focusing on the electrolyte). The typical anions we will introduce are displayed in Fig. 1, accompanied by the corresponding donor number (DN), which is a well-known parameter representing the coordination ability. By comprehensively understanding and fully utilizing the functions of anions in electrolytes, the electrochemical systems can be better designed and optimized, improving electrochemical performance, cycle life, and safety of the battery, which will make great contributions to promoting sustainable energy conversion and application. The following sections will delve deeper into the applications of anions in electrolytes, as well as their potential significance and future research directions.

## Impact of Anions on the Interface

The SEI has a significant impact on the battery performance [43–47]. However, the formation of this interface is very complex and lacks in-depth research. The decomposition of anions plays a crucial role in the formation of the SEI layer [28, 48]. Before the formation of the SEI, a double layer and charge transfer are formed and processed at the interface, followed by chemical reactions and deposition of organic/inorganic components [49]. Next, we will explore how anions can adjust the interface from three aspects. The first is the anion-induced SEI formation, which mainly includes the formation of CEI at the cathode, the formation of AEI at the anode, as well as the formation of cathode electrolyte interphase (CEI) and anode electrolyte interphase (AEI) at the cathode and anode. The second is the detailed elaboration of how the anion repairs the SEI repair as well as the mechanism, and the last is the in-depth discussion of anion-regulated EDL.

### Anion-Induced SEI

A SEI is derived from the decomposition of anions and solvents in the electrolyte. So, the electrolyte components play a curial role in the SEI properties [28, 50, 51]. The inorganic components (such as LiF and Li_3_N) in SEI are usually considered good for the rigidity of this SEI and for a fast Li-ion transition [52]. It is known that inorganic components directly/indirectly come from the anions. Therefore, this section focuses on the anion-induced SEI, discussing the effect of anions on the formation of AEI, CEI, and both the AEI and CEI.

#### Anion-Induced AEI

From the anode side, the application of lithium bis(fluorosulfonyl)imine (LiFSI)-based carbonate electrolytes (1 M LiFSI dissolved in ethylene carbonate (EC) and dimethyl carbonate (DMC) (1:1 v/v)) in LIBs has been reported to offer better cycling performance than traditional LiPF_6_-based electrolytes [27]. This improvement is attributed to FSI^–^, which contributes to forming a dense AEI film that can protect the graphite anode. Zhang et al. proposed a dual-salt electrolyte consisting of 0.8 M LiFSI and 0.2 M lithium difluoro(oxalato)borate (LiDFOB) in fluoroethylene carbonate (FEC)/tetra (ethylene glycol) dimethyl ether (TEGDME) [53]. In this electrolyte, the addition of LiDFOB effectively prevents the dissolution of Fe from the LiFePO_4_ cathode [54]. Moreover, both LiFSI and FEC facilitate the formation of a thermally stable AEI on the surface of the graphite anode, particularly at high temperatures (70 °C), significantly enhancing the safety of the batteries.

**Fig. 2:**
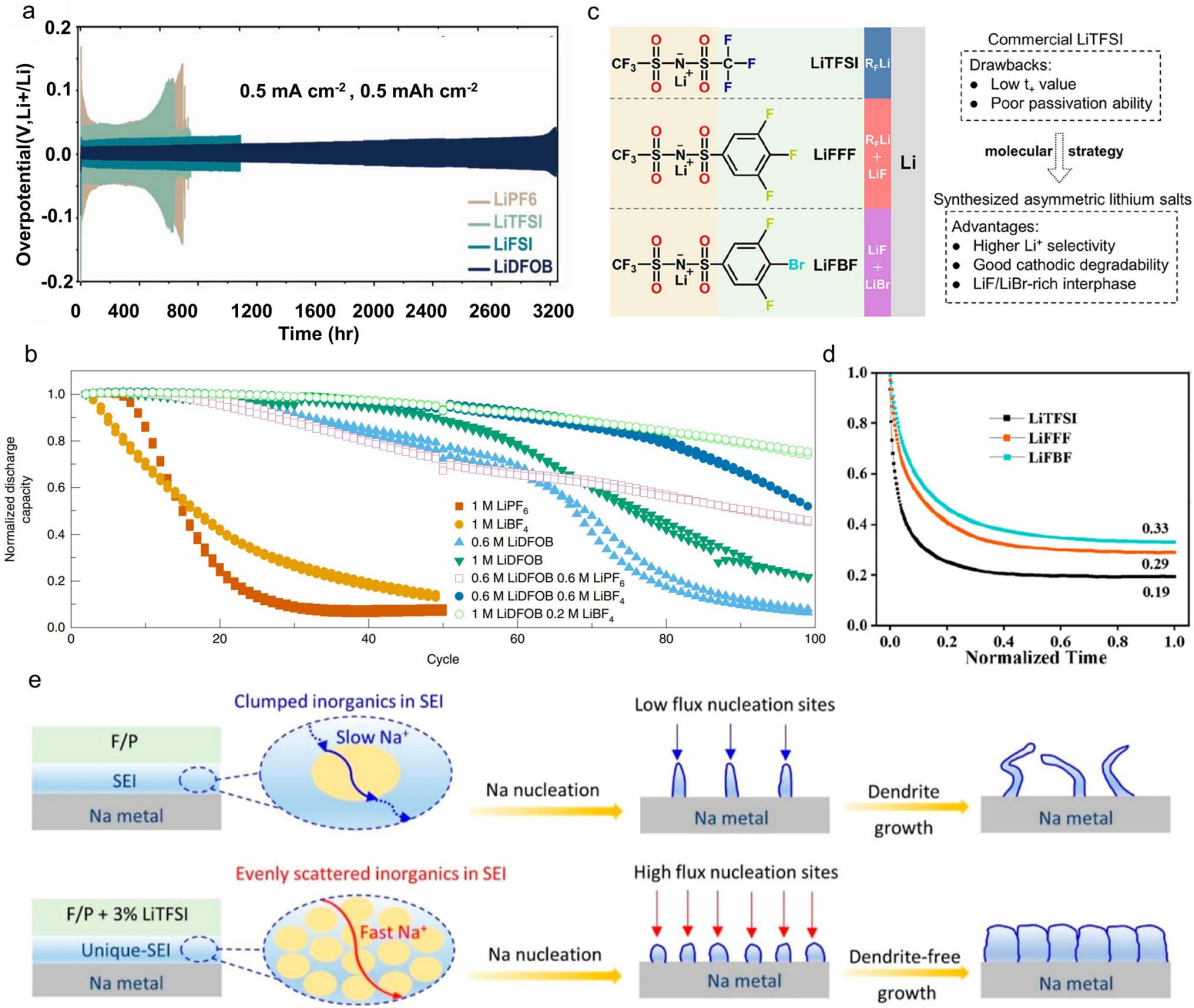
**a** Long-term stability testing of Li||Li symmetrical cells using various THF-mixed electrolyte systems under low current density conditions [25]. Copyright 2023, Wiley–VCH GmbH. **b** The relationship between cycle number and capacity retention for anode-free pouch cells with various lithium salt electrolytes [55]. Copyright 2019, Springer Nature. **c** Two novel lithium salts with structures and film formation properties based on LiTFSI. **d** Li^+^ transference number of the polymer electrolytes with different Li-salts [56]. Copyright 2023, American Chemical Society. **e** Schematic diagram illustrating the SEI formation in electrolytes with and without LiTFSI [57]. Copyright 2023, American Chemical Society

In addition to the formed AEI on the graphite, Mullins et al. utilized various lithium salts (e.g., LiPF_6_, LiTFSI, LiFSI, and LiDFOB) to achieve high stability lithium anodes based on tetrahydrofuran (THF) electrolyte systems [25]. The anions preferentially decompose instead of organic solvents, producing inorganic-rich AEI. The LiDFOB-based electrolyte has the lowest viscosity (1.10 mPa s), highest Li^+^ transport numbers, and highest ionic conductivity (16.1 mS cm^−1^) among the electrolytes based on different Li-salts. Moreover, the AEI film generated by the decomposition of DFOB^–^ is the thinnest, which may be the reason that the LiDFOB electrolyte offers an excellent cycling performance (over 3200 h) in a symmetrical cell at a current density of 0.5 mA cm^−2^ (Fig. 2a). Dahn applied LiDFOB and LiBF_4_ as dual-salt carbonate electrolyte additives in an anode-free lithium pouch cell, which maintains 80% capacity retention after 90 cycles [55]. However, the stability of the cell with single-salt electrolyte (LiBF_4_) drops below 80% retention in less than 15 cycling cycles (Fig. 2b). The AEI film established within the dual-anions contains a significant quantity of LiF, which promotes the uniform deposition of lithium. In addition to some commonly used anions, there are also some new lithium salts (anions) that can also modify the interface. For example, Liu et al. developed an asymmetric trihalogenated aromatic lithium salt, namely LiFFF and LiFBF [56] (Fig. 2c), as a single conductive lithium salt in polymer electrolytes for all-solid-state lithium batteries. The electrolytes with LiFFF and LiFBF not only possess high Li^+^ transport number (Fig. 2d) but also generate a good AEI on the negative electrode surface. Particularly, LiFBF induces a LiF/LiBr mixed AEI that can passivate the lithium metal anode because LiF exhibits a high Young’s modulus and high surface energy to homogenize Li^+^ flux, while the activation energy for ion transport in the interphase of LiBr is reduced by approximately three times compared to LiF. Therefore, the interface stability of the anode has been significantly enhanced. As a result, Li||Li symmetric cell can stably cycle for over 1200 h without short circuits. And Li||LiFePO_4_ batteries can work for 1200 cycles at rate of 1 C, demonstrating excellent electrochemical performance.

**Fig. 3:**
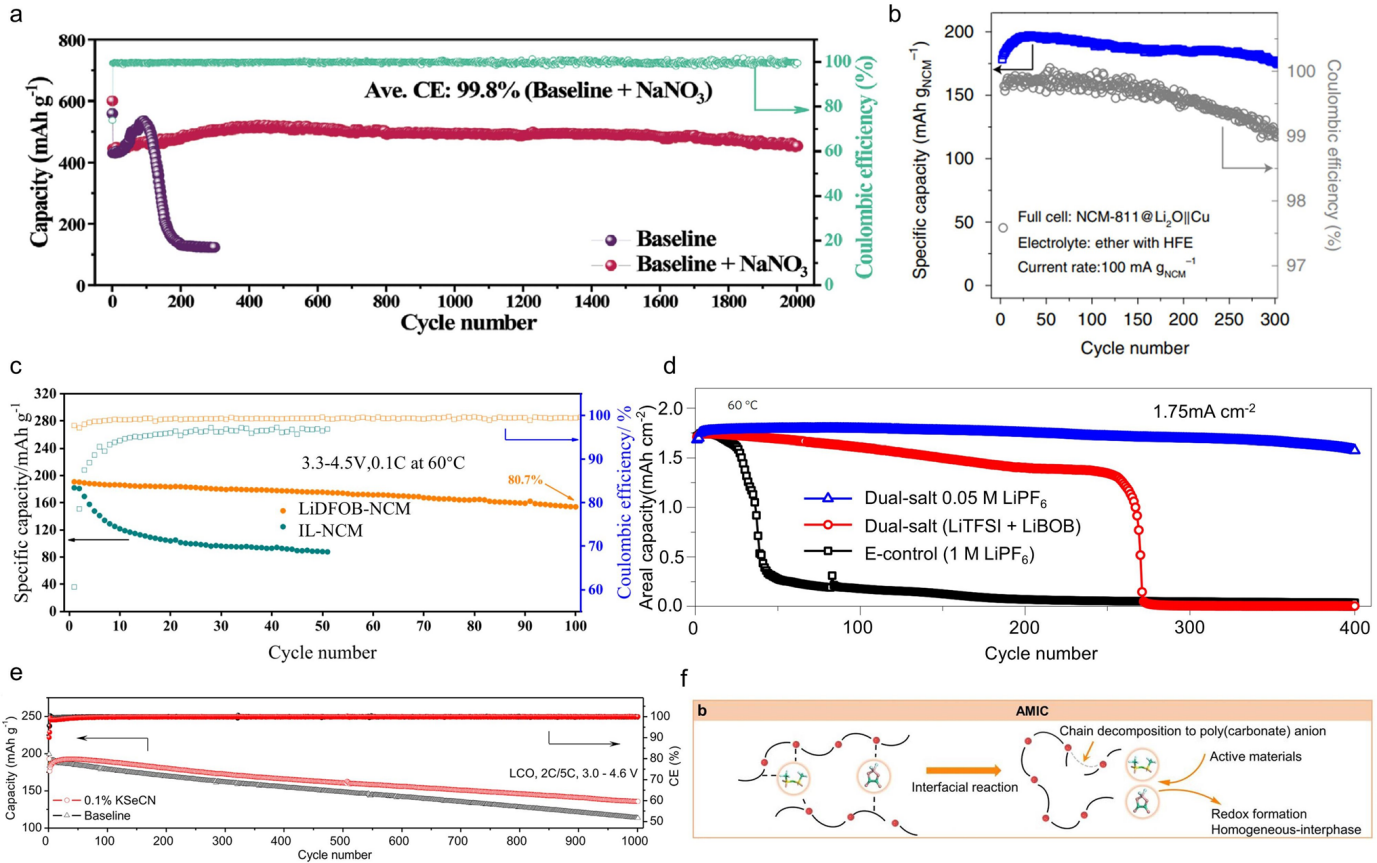
**a** Cycle performance of the Na||FeS@NS-C half cells using baseline and optimized electrolytes at 1.0 A g^−1^ [58]. Copyright 2023, Wiley–VCH GmbH. **b** Discharge capacity hysteresis plotted against cycle number [5]. Copyright 2021, Springer Nature. **c** Cycle performance in the voltage range of 3.3–4.5 V with and without a dual-salt composite cathode [59]. Copyright 2023, Elsevier. **d** Cycling performance of Li||NMC batteries with different electrolytes at 60 °C [60]. Copyright 2017, Springer Nature Limited. **e** Cycling performance of Li∥LCO batteries using baseline and optimized electrolytes at a 2C charge rate and a 5C discharge rate [61]. Copyright 2022, American Chemical Society. **f** Schematic representation of the interfacial decomposition mechanism of anion-modulated ion conductor (AMIC) [63]. Copyright 2024, Wiley–VCH GmbH

Like lithium batteries, anion chemistry has also been used in sodium batteries to regulate the interphase. Chen et al. constructed a unique uniformly dispersed high conductivity inorganic-rich AEI by introducing self-sacrificial LiTFSI into sodium-based electrolytes (0.75 M NaClO_4_ in FEC/PC (propylene carbonate)) [57]. The reduction between LiTFSI and FEC promotes the formation of inorganic SEI (Fig. 2e). Among them, highly conductive inorganic substances provide fast ion transport domains and high-throughput nucleation sites for Na^+^, which is conducive to the rapid deposition of sodium. The AEI derived from LiTFSI and FEC resulted in high capacity retention of 89.15% in the Na||Na_3_V_2_(PO_4_)_3_ battery after 1000 cycles at an ultra-high rate of 60 C. Xia et al. introduced NaNO_3_ into the ester-based electrolyte (1 M NaClO_4_ in EC/PC (1:1) with 2% FEC) to control interface chemistry and AEI properties [58]. Due to the strong solvating ability of NO_3_^–^, the NO_3_^–^ occupies the solvation shell of Na^+^ and decomposes instead of other anions. With the help of FEC additives, a stable AEI containing Na_3_N and NaF is generated on Na metal surface. With this advantageous AEI, the lifespan of electrodes based on transition metal sulfides (FeS@NS-C) can be markedly prolonged by more than 2000 cycles (Fig. 3a).

#### Anion-Induced CEI

Zhou et al. introduced Li_2_O as a preloaded sacrificial agent mixed with LiNi_0.8_Co_0.1_Mn_0.1_O_2_ cathode to provide an additional lithium source to offset the irreversible loss of lithium for long-term cycling of batteries [5]. In situ surface-enhanced Raman spectroscopy (SERS) studies have shown that the O_2_^–^ anions released through the oxidation of Li_2_O are synergistically neutralized by the fluorinated ether additives, forming a LiF-based CEI, which passivates the surface of the cathode and inhibits the deleterious oxidative decomposition of the solvent. As a result, a long-life 2.46-Ah anode-free pouch battery was developed with a gravimetric energy density of 320 Wh kg^−1^ retaining 80% capacity retention after 300 cycles (Fig. 3b). Xie et al. reported a strategy by adding a trace amount of LiDFOB to the NCM811 cathode to form a high-voltage solid-state lithium metal batteries (LMBs) [59]. A CEI layer consisting of LiF and Li_*x*_BO_*y*_F_*z*_ was in situ constructed through the synergistic decomposition of DFOB^−^ and TFSI^−^ from ionic liquids, which effectively suppressed the occurrence of side reactions and cracks of NCM particles during the high-voltage cycles. Therefore, the solid-state lithium battery with CEI-protected NCM811 exhibits an initial specific capacity of 190.7 mAh g^−1^ at a high cut-off voltage of 4.5 V at rate of 0.1C and capacity retention of more than 80% at 60 °C for 100 cycles, while the capacity of the cell without adding LiDFOB at a high voltage of 4.5 V dropped to 48.3% after only 50 cycles (Fig. 3c). Wu et al. added a trace of LiPF_6_ (0.05 M) additive to a dual-salt (LiTFSI-LiBOB) carbonate electrolyte, vastly improving the cycle stability of LMBs (Fig. 3d). The addition of LiPF_6_ serves two purposes: the one is that it can stabilize the Al current collector, suppressing the Al corrosion. More significantly, a small amount of LiPF_6_ additively changed the component of the SEI. The improved interfacial stability facilitates the continuous operation of LMBs even at high charge current densities [60].

#### Anion-Induced AEI and CEI

Zheng et al. reported a novel electrolyte additive of potassium selenocyanate (KSeCN) for LMBs [61]. The additive has a high HOMO (the highest occupied molecular orbital) and a low LUMO (the lowest unoccupied molecular orbital), allowing it to preferentially decompose during battery charging and discharging processes. It forms a Se-containing protective layer on the surface of the LiCoO_2_ (LCO) cathode and lithium metal anode, thereby inhibiting the decomposition of the electrolyte at the cathode/anode interface. The functional additive can retard the dissolution of transition metal ions and significantly improve the stability of the lithium metal anode. Compared with Li||LCO battery with traditional ester electrolytes, the battery with 0.1 wt% KSeCN additive can maintain stable cycling at a high cut-off voltage of 4.6 V (Fig. 3e). Xia et al. regulated the carbonate-based electrolyte through the multi-functional additive lithium difluorobis(oxalato) phosphate (LiDFBOP) [62]. Theoretical calculation and physical and chemical characterization confirmed that DFBOP^–^ as film forming agent, preferentially reacted on both sides of the Na_3_V_2_(PO_4_)_2_F_3_ cathode and Na anode to form a stable and strong interphase (CEI/AEI). At the same time, Li^+^ regulates the stability of the electrolyte by regulating the solvation structure and suppresses dendritic growth through electrostatic shielding.

Fan et al. proposed a strategy called anion-modulated ionic conductor (AMIC) to address the problem of uneven electrolyte–electrode interface stability in solid-state LMBs [63]. By employing two types of anions (DFOB^–^ and TFSI^–^), the decomposition behavior of the polymer chain segments at interphase was altered, converting poly (vinylene carbonate) (PVC) to polycarbonate anion, which promotes the uniform deposition of lithium ions (Fig. 3f). Specifically, the polymer chain segments shift to a more favorable entropic globular conformation under the influence of anions (coordination properties of carbonyl groups). In addition, the spherical conformation exposes more lithium-ion binding sites, improving the interchain transfer of lithium ions [64]. Consequently, the electrolyte exhibits high ionic conductivity of 1.78 × 10^−4^ S cm^−1^ (at 60 °C), high Li^+^ transport number (*t*_Li_^+^ = 0.67), and a broader electrochemical window (upon to 4.8 V). Furthermore, the two anions form stable SEI on both sides. The AEI maintains uniform deposition of Li-ions and inhibits dendric Li growth, while the CEI preserves the structural stability of the cathode. Li || LiNi_0.8_Co_0.1_Mn_0.1_O_2_ cell maintains about 85% capacity retention and the coulombic efficiency is > 99.8% in 200 cycles at a charge cut-off voltage of 4.5 V.

### Repairing SEI by Anions

The failure of LMBs is caused by various reasons, including the growth of dendritic lithium, accumulation of inactive lithium (also known as dead lithium), and unstable SEI [65, 66]. The presence of dead lithium is the primary cause of the performance degradation in LMBs [67]. There is an urgent need for a basic solution for recycling dead lithium to stabilize LMBs [68].

**Fig. 4:**
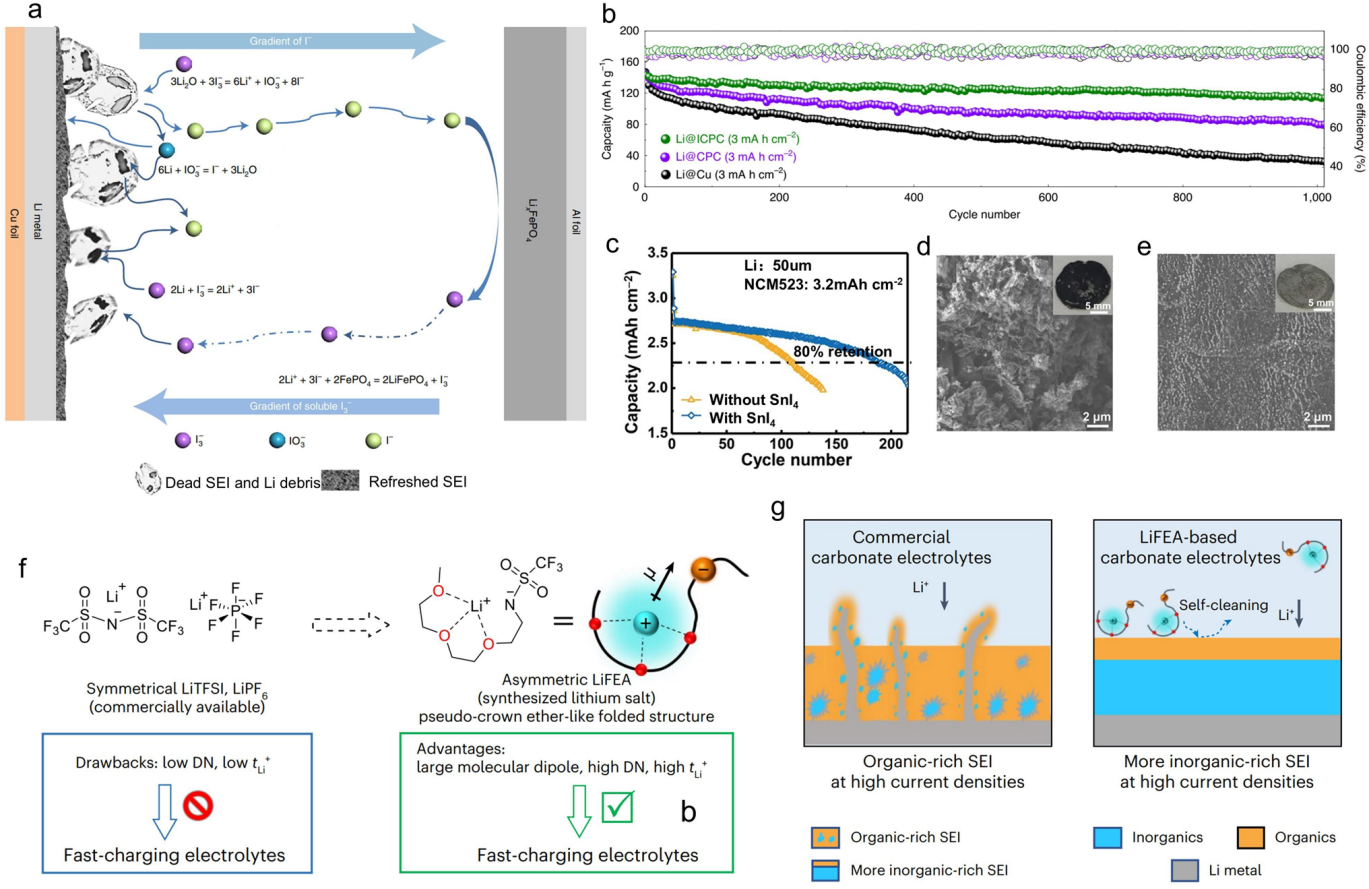
**a** Schematic diagram of the mechanism for lithium restoration based on iodine redox cycling. **b** Cycling performance of Li@ICPC, Li@CPC and Li@Cu anodes with a low N/P ratio of 2.5 in LFP full cells at 1C [69]. Copyright 2021, Springer Nature. **c** Cycling performance of Li||NCM523 without SnI_4_ and with SnI_4_. **d** SEM image of Li foil after cycling in LiPF_6_-DMC/FEC electrolyte. **e** SEM image of cycled Li foil after soaking in LiPF_6_-DMC/FEC electrolyte with 4.0 mM SnI_4_ additive [31]. Copyright 2021, Wiley–VCH GmbH. **f** Comparison of LiFSI, LiPF_6_ with LiFEA. **g** Schematic representation of LiFEA-based electrolytes enabling a more inorganic-rich SEI layer and dendrite-free Li deposition through self-cleaning action compared to commercial carbonate electrolytes [72]. Copyright 2023, Springer Nature

Tao et al. proposed a lithium reduction method based on a series of iodine redox reactions primarily involving I_3_^–^/I^–^ process [69]. Using iodine as a biochar capsule host, they found that the I_3_^–^/I^–^ redox takes place spontaneously. As shown in Fig. 4a, LiI, as an intermediate product, functions as a carrier for the lithium element (Li-vehicle), while LiIO_3_ as a carrier for the oxygen element (O-vehicle). The reaction can effectively activate “dead lithium” and facilitate the transport of Li_2_O within the “dead SEI”. Meanwhile, Frontline molecular orbital theory calculations confirm that iodine species exhibit a higher electron affinity compared to solvent molecules and anions (of Li-salts) in the electrolyte, making iodine preferentially react with lithium metal, thereby inhibiting the decomposition of the electrolyte. With this design, a full battery assembled with a Li@ICPC (iodine-carbonized porous conidial powder) anode and LiFePO_4_ (LFP) cathode can even maintain 1000 cycles with capacity retention of 80% under a low negative/positive (N/P) ratio of 2.5 (Fig. 4b). Zhang et al. also recovered inactive lithium by using an I_3_^–^/I^–^ redox pair initiated by stannic iodide (SnI_4_) [31]. The I_3_^−^ facilitates the conversion of inactive Li into soluble LiI, which then migrates toward the cathode. Following this, the delithiated cathode oxidizes LiI, regenerating I_3_^–^ through cathode lithiation. Consequently, inactive Li is continuously reclaimed during the battery cycling. The viability of this strategy for recycling inactive lithium has been successfully demonstrated in Li||LiNi_0.5_Co_0.2_Mn_0.3_O_2_ batteries, resulting in a doubled lifespan (Fig. 4c). In addition, the feasibility of the method was further verified by comparing the morphology of lithium after cycling (Fig. 4d, e). This method is also applicable to solid polymer electrolytes (SPE). Tao et al. applied a trace amount of I_2_ into polyethylene oxide (PEO) electrolytes to establish stable interphase on lithium metal surfaces, thus ensuring prolonged battery cycling [70]. The I_3_^–^ from iodine additives can coordinate with the − COC − bond of PEO, thereby improving the ionic conductivity of the SPE. In addition, I_3_^−^ can spontaneously react with dead Li and Li_2_O, leading to the formation of a dense and uniform iodine-doped SEI layer in situ on the lithium anode surface, ultimately reducing interface resistance and inhibiting dendrite growth. Li et al. synthesized iodine-doped polyacrylonitrile (I-PAN) negative electrode materials using a low-temperature calcination method, demonstrating an excellent fast-charging performance of lithium/sodium-ion batteries [71]. The iodine of the I-PAN backbone is prone to nucleophilic substitution reaction with the PF_6_^−^ in the electrolyte to form I^−^. Raman/NMR/molecular dynamics simulations show that I^−^ readily enters the solvated inner layer of Li^+^ and promotes its desolvation process. Under fast-charging conditions, the dead lithium formed on the negative electrode can be reused through reversible I^−^/I_3_^−^ redox reactions to compensate for the lithium loss. Therefore, I-PAN enables rapid bulk/interface Li^+^ diffusion kinetics, accelerated desolvation processes, formation of SEI rich in LiF/LiI, and reuse of dead lithium. When discussing the issue of dead lithium recovery, the stability of the SEI and its crucial role in the lithium deposition and stripping processes cannot be overlooked. The formation of dead lithium is closely related to the failure of the SEI, and the success of dead lithium recovery also depends on effective SEI repair. By repairing the SEI, lithium dendrite growth can be suppressed, which in turn reduces the generation of dead lithium. Therefore, dead lithium recovery and SEI repair are not only interrelated but also collaborative improve the cycling stability and lifespan of lithium metal batteries. Next, we will further explore the specific mechanisms of anion-based SEI repairing and its applications in electrolyte design.

Specific Li-salts can also be used for self-repairing of SEI. An asymmetric Li-salt, lithium 1,1,1-trifluoro-N-[2-[2-(2-methoxyethoxy) ethoxy)] ethyl] methanesulfonamide (LiFEA) was designed by Liu et al. (Fig. 4f), which has a pseudo-crown ether-like folded molecular geometry and gives carbonate electrolytes, high large apparent donor number and Li^+^ transference number [72]. More importantly, during the SEI construction process, the anion can dissolve unwanted organic species (for example, ROCO_2_Li, -(CH_2_CH_2_O)_*n*_-) formed in carbonate electrolytes (Fig. 4g), leading to the gradual enrichment of inorganic species in the SEI. They combined quartz crystal microbalance analysis with electrochemical methods (EC-QCM) to monitor changes in SEI quality during immersion treatment. The SEI mass decreased by 25.7 μg, which is three times higher than that of the blank case (1 M LiPF_6_ EC/DEC (v/v = 1:1)). This finding is consistent with the X-ray photoelectron spectroscopy (XPS) and time-of-flight secondary ion mass spectrometry (TOF–SIMS) results from the soaking experiment. This unique self-cleaning mechanism helps to generate dense and uniform SEI rich in inorganic substances. Based on LiFEA-based carbonate electrolyte, the Li||NCM811 battery exhibits outstanding cycling performance. The pouch cell maintained 81% capacity retention after 100 cycles (charge: 0.4C, discharge: 1C). In contrast, the blank cell survived only 50 cycles under identical conditions. These results indicate that LiFEA-based carbonate electrolyte has promising potential for widespread application in high-performance LMBs. However, through in-depth research into the physicochemical properties of LiFEA, it has been discovered that the excessively high donor number (DN) of FEA^−^ can lead to high reactivity and poor compatibility with some solvents (such as FEC). Therefore, to address this issue, a new lithium salt lithium, 2-[2-(2-methoxy ethoxy)ethoxy]ethanesulfonyl(trifluoromethanesulfonyl) imide (LiETFSI) has been introduced based on FEA^−^ by incorporating sulfonyl groups [73]. Electrolytes containing LiETFSI in carbonate solvents not only ensure high conductivity but also exhibit a higher Li^+^ transference number (*t*_Li_^+^≈0.8) with a moderate DN. The anion facilitates the dissolution of oxygen-containing organic compounds in the SEI while reducing the dissolution of inorganic components such as LiF, confirmed by XPS and TOF–SIMS. Consequently, the electrolyte establishes a SEI interface with a lower impedance rich in inorganic components. The advanced LiETFSI electrolyte (with LiNO_3_) enhances the CE to 99.0%, indicating a high reversibility of the Li plating/stripping process. In comparison, the CE with advanced LiFEA electrolyte only reaches 96.6%, and the addition of FEC does not make a big difference due to the poor compatibility between LiFEA and FEC. Taking advantage of the film-forming properties of FSI^−^ and the photopolymerization properties of vinyl, Wang et al. crosslinked the ionic liquid 1-vinyl-3-methylimidazolium bis(fluorosulfonyl)imide (VMI-FSI) with poly(ethylene oxide), to form a self-healing membrane with the FSI^−^ group as the repair agent [74]. When they encounter lithium metal, the FSI^−^ groups are chemically decomposed into LiF and Li_3_N, which assist in forming SEI on lithium metal and repairing SEI in the cracks lacerated by lithium dendrite. Furthermore, the FSI^−^ anions exchanged from the film are electrochemically decomposed to inorganic components to strengthen the SEI. This self-healing layer demonstrated excellent performance in Li||LCO cells which are stably operated for 500 cycles with the retention rates of 81.4% and the average coulombic efficiency of 99.97% at 4.5 V. The strategies hold significant potential for applications in the field of LMB. Although researchers have proposed methods based on iodine-based redox reactions and designing self-healing molecules to repair dead lithium and SEI, in practical applications, these approaches may encounter challenges such as stability and cost issues. Therefore, it remains imperative to consider how to address such a challenge.

### Anion-Modulated EDL of the Cathode

**Fig. 5:**
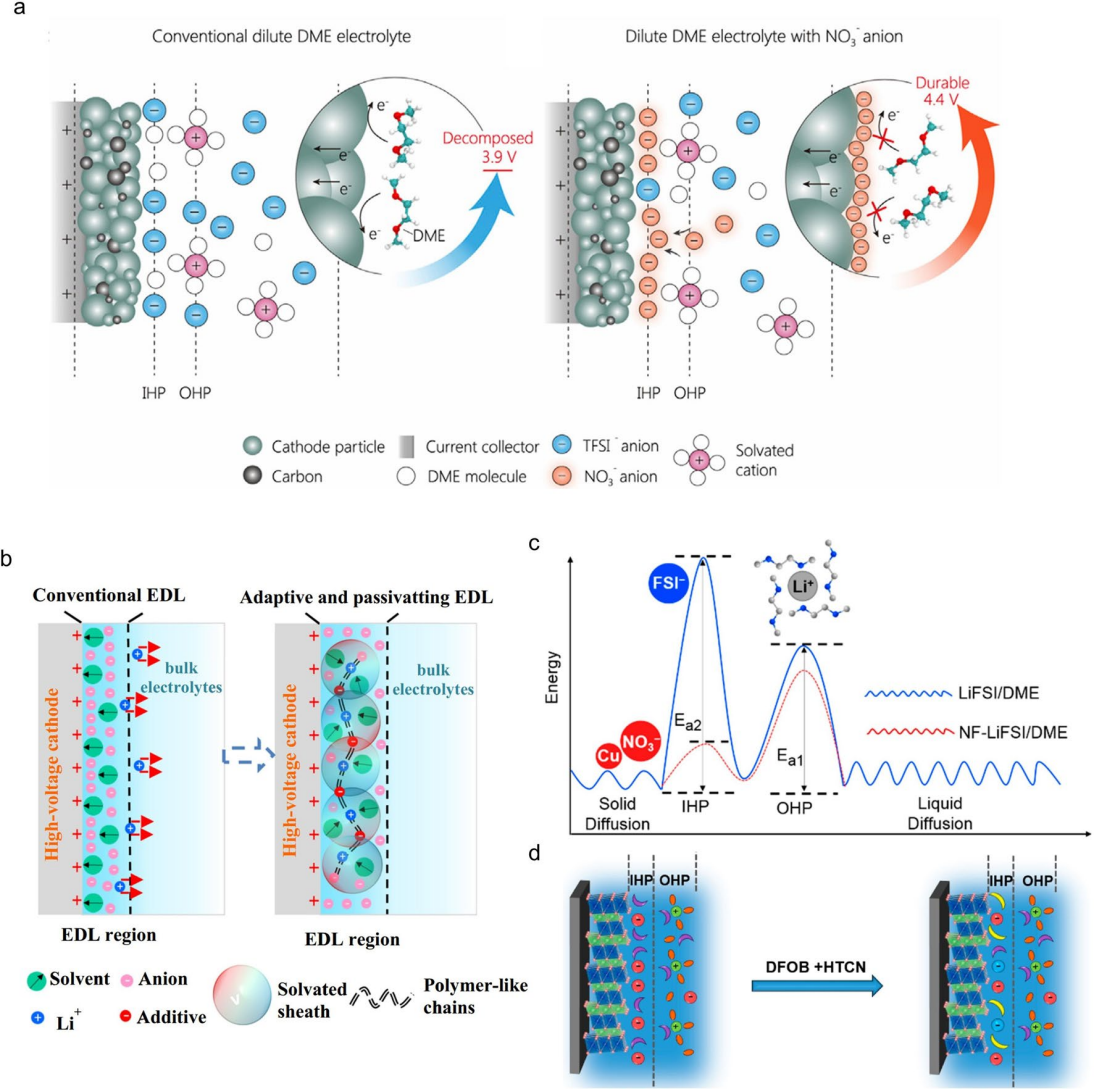
**a** Schematic representation of the mechanism for improving and optimizing the interface by adding nitrate ions to the traditional ether-based dilute electrolyte [33]. Copyright 2022, Elsevier. **b** Proposed mechanism for constructing a dynamic high-voltage resistance EDL [34]. Copyright 2022, Springer Nature. **c** Correlation between the energy barrier of Li^+^ and the specific adsorption in the inner Helmholtz plane (IHP) and outer Helmholtz plane (OHP) [74]. Copyright 2019, American Chemical Society. **d** Schematic illustration of IHP changes after adding additives of HTCN and LiDFOB [76]. Copyright 2023, American Chemical Society

The electrochemical reactions take place in the EDL, actually in the inner Helmholtz plane (IHP), and therefore, the evolution of EDL at the electrode/electrolyte interface is very critical [74, 75]. The characteristics of this layer on the cathode surface can be modulated by the anions due to the electric field in the IHP. Herein, the interface properties of the cathode have a directly relationship with the anions. The anion in the IHP that can regulate the CEI formation process is optimal, so as to prevent excessive electrolyte consumption, especially for the ether electrolytes. Kang et al. proposed an electrolyte design strategy to enhance the oxidation stability of a dilute ether electrolyte (1 M LiTFSI in DME) using trace amounts of nitrate additive (50 mM NO_3_^−^) [33]. The combined experiments and molecular dynamics simulations reveal that a novel interface is formed by the aggregation of NO_3_^−^ anions in the IHP, thereby eliminating solvent molecules at the interface and inhibiting the oxidative decomposition of solvent molecules (Fig. 5a). This adsorption behavior is crucial for expelling solvent molecules at the interface and regulating the electrochemical environment of the interface. As a result, the ether electrolyte can endure a high voltage of 4.4 V. Meanwhile, Liu et al. also applied NO_3_^−^ additives to ether-based electrolytes (1 M LiFSI in DME) to construct a dynamic and highly stable EDL at the CEI [34]. This non-traditional EDL is filled with nitrate ions, enhancing the oxidation stability of ether-based electrolytes and passivating the decomposition of the electrolyte. Specifically, NO_3_^−^ can quickly migrate to the cathode interface under the electric field during the battery charging, forming a “supramolecular dynamic network structure” with Li^+^ and solvents (Fig. 5b). With the improved EDL, LMBs utilizing conventional ether electrolytes (≤ 1 M) exhibit excellent performance, such as high cut-off voltage up to 4.5 V (vs. Li/Li^+^), excellent ultrafast cycling performance (Li||NCM811, 5 C, 90% capacity retention after 700 cycles), and ultra-low temperature performances (86 mAh g^–1^ at –91 °C). In addition, Zhang et al. used trace amounts of LiNO_3_ and CuF_2_ in ether-based electrolytes to modify the structure of the EDL [74]. They verified that NO_3_^−^ is more easily adsorbed as the dominant anion in the IHP compared with FSI^–^ (Fig. 5c). The special structure ensures a good SEI containing nitrogen oxides, inducing spherical growth of metallic lithium. The lifespan of Li||LiFePO_4_ cell (capacity retention of 92.5% after 700 cycles) is at least 12 times than that of the batteries without the additives.

In addition to applying NO_3_^−^, Lu et al. proposed a specific adsorption-oxidation (Ad-O) strategy. The formation of the CEI is determined by utilizing trace amounts of 1,3,6-hexanetricarbonitrile (HTCN) and LiDFOB (Fig. 5d) [76]. The HTCN and DFOB^−^ compete with the primitive carbonates and PF_6_^−^ for adsorbing at the cathode surface by specific electron-rich groups to form novel structures within the IHP, thereby “squeezing” the most primitive compositions. This strong interaction masks the active sites on the surface of the cathode, preventing a weak CEI produced by the dehydrogenation and oxidation of traditional carbonates. After initial cycles, a double-layer CEI was formed with LiF as the inner layer and organic-rich B − F/ − CN component as the outer layer, further protecting the electrolyte and the cathode from decomposition and crystal degradation, respectively. This cooperative effect makes the cathode maintain stable cycling performances even under high voltages of 4.6 V and elevated temperatures of 60 °C.

Chen et al. improved the storage performance of Na_2.26_Fe_1.87_(SO_4_)_3_ by exposing the (112) plane to constitute a robust CEI [77]. The special plane promotes the adsorption of the ClO_4_^−^ and FEC, forming an inorganic-rich Na^+^ conductive interphase at the cathode. As a result, when tested in combination with a presodiated FeS/carbon-based negative electrode in laboratory-scale single-layer pouch cell configuration, the Na_2.26_Fe_1.87_(SO_4_)_3_-based positive electrode enables an initial discharge capacity of about 83.9 mAh g^–1^.

Researchers have also proposed the application of lithium trifluoroacetate (LiTFA)-LiNO_3_ dual-salt additives in LiPF_6_-based carbonate electrolytes [78]. By constructing a thermodynamically favorable EDL structure on the positive electrode interface, the kinetics of interfacial reactions have been significantly accelerated. This greatly enhances the thermodynamic compatibility between the carbonate electrolyte and the high-voltage layered transition metal oxide positive electrode. Specifically, the use of hygroscopic LiTFA as a dual-functional additive inhibits the hydrolysis of LiPF_6_ and promotes the dissolution of LiNO_3_ in the carbonate electrolyte. Even after exposure to humid air for 12 h, the capacity retention of 78.3% can be achieved after 200 cycles in Li||NCM523 batteries using a newly proposed moisture-resistant carbonate electrolyte.

## Influence of Anions on the Solvation Structure

The solvation structure refers to the arrangement formed when solute ions are surrounded by solvent molecules in an electrolyte solution [79]. This structure directly affects the movement and reactivity of ions in the solution [80]. Solvent molecules interact with solute ions through electrostatic interactions, hydrogen bonding, or coordinate bond, resulting in the formation of a solvation shell [28]. The composition, structure, and physicochemical properties of SEI largely depend on the solvation structure of the electrolyte. To modulate the solvation chemistry in liquid electrolytes, it is imperative to comprehend the interplay between ions and solvents. These interactions are intricately linked to regulating interface stability, expanding the electrochemical stable potential window, controlling kinetics, and suppressing side reactions within batteries.

### Anion-Regulated Stable Interface

As discussed above, SEI is formed through the decomposition of the anions and solvents. Therefore, the SEI properties can be controlled by tuning the electrolyte components (solvation structure). Zhang et al. proposed that when the anion is coordinated with the Li^+^-solvent complex, the LUMO energy level of the solvent becomes higher and that of the anion becomes lower [81]. This indicates that the participation of anions in the Li^+^-solvent sheath not only improves the reduction stability of the solvent but also promotes the reduction of the anions. Anions in the solvation sheath readily accept electrons and undergo a reduction process, leading to the formation of an inorganic-rich AEI. The use of anions with high DN values is often an effective strategy for modulating the solvation sheath [82]. As an example, LiNO_3_ is also a well-known film-forming agent with a high DN value (22 kcal mol^−1^) and is usually used in electrolytes as an additive [15, 83–85]. Introducing NO_3_^−^ into the electrolyte can obtain a solvation structure rich in NO_3_^−^ while poor in solvents, inducing a large number of contact ion pairs (CIPs) and ion aggregates (AGGs). The solvation structure regulated by NO_3_^−^ dominates the formation of inorganic-rich SEI [86].

**Fig. 6:**
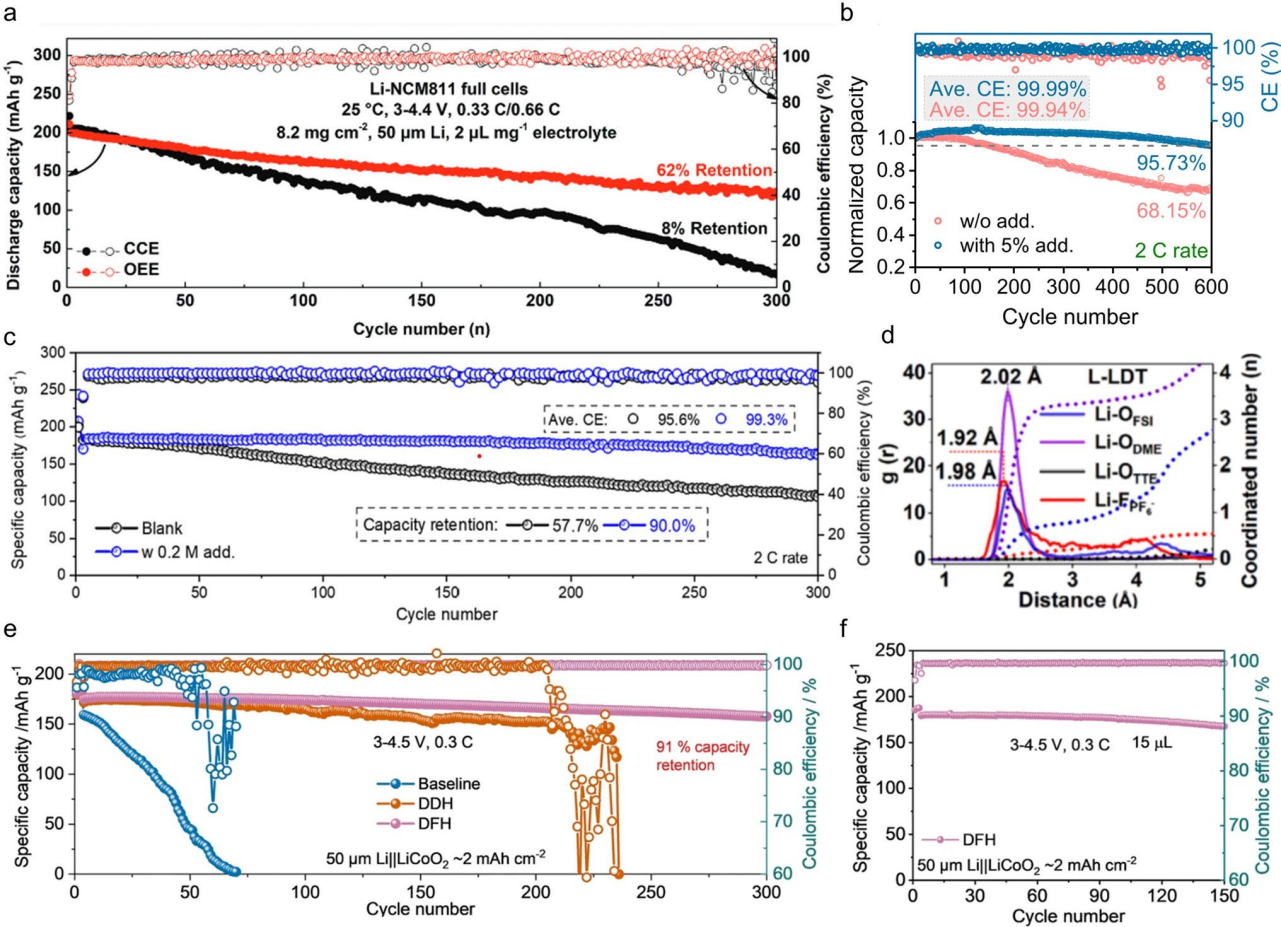
**a** Comparison of cycling performance of Li-NCM811 full cell using different electrolytes at 25 °C (OEE:1 M LiDFOB in DME + 0.5 M LiNO_3_ + 5 vt% VC; CCE: 1 M LiPF_6_ in EC/DEC) [88]. Copyright 2023, Wiley–VCH GmbH. **b** Cycling performance and CE based on normalized capacity at the 2C rate in 1 M LiPF_6_ in EC/DEC with and without Cu(NO_3_)_2_ additive [89]. Copyright 2021, American Chemical Society. **c** The electrochemical performance of Li|NCM811 cell with different electrolytes between 3.0 and 4.3 V at 2C rate [91]. Copyright 2022, Elsevier. **d** The corresponding RDF of the Li-O_DME_, Li-O_TTE_, Li-F_PF6_, and Li-O_FSI_ pairs in 1.0 M LiFSI and 0.1 M LiPF_6_ electrolytes in DME-hydrofluoroether (TTE) (with Li^+^: DME = 1: 4) [92]. Copyright 2022, Elsevier. **e** Long-term cycling performance of Li||LCO cells using baseline, DDH, and DFH electrolytes. **f** Cycling performance of Li||LCO cells using DFH with limited conditions [20]. Copyright 2022, Springer Nature

Chen et al*.* introduced LiPF_6_ and LiNO_3_ as dual-salt additives in an electrolyte of LiTFSI in DOL/DME and obtained a high CE of 99.3% and long cycling stability of the Li||LFP (*N*/*P* = 2) cell [87]. The enhancement of the cell could be attributed to the formation of a good SEI that contains both inorganic and organic components. Li et al. developed an optimized ether-based electrolyte (OEE) by adding LiNO_3_ and vinylene carbonate (VC) [88]. This electrolyte (1 M LiDFOB and 0.5 M LiNO_3_ in pure DME with 5 vt% VC) provides high lithium-ion conductivity (11.52 mS cm⁻^1^ at 20 °C) and high voltage stability (4.4 V). LiNO_3_ and VC can enter the inner solvation shell and preferentially participate in the film-forming process at the electrode surface, resulting in the formation of a unique organic–inorganic bilayer interfacial protective layer. This protective layer effectively suppresses electrolyte side reactions and enhances electrode stability. Consequently, the 4.4 V NCM811 full cells assembled with the OEE exhibit stable cycling performance at both room temperature (Fig. 6a) and low temperatures (− 20 °C).

The solubility of most nitrates in carbonate electrolytes is rather limited, so in previous work, Lewis acid was used as an electron-rich NO_3_^−^ acceptor to decompose LiNO_3_ clusters [89, 90]. For example, our previous work used Cu(NO_3_)_2_ as an additive to regulate the solvation structure of 1 M LiPF_6_ in EC/DEC (1:1, vol%) electrolyte. Since the electrostatic interaction of Li^+^ with NO_3_^−^ is stronger than with PF_6_^−^, NO_3_^−^ entered the solvation sheath of Li^+^, which can be demonstrated by NMR and Raman results. XPS results showed that the inorganic-rich components in the SEI, which is favorable for the Li conduction and the stability of the SEI. The Li||NCM811 cell exhibits capacity retention as high as 95.73% after 600 cycles at room temperature (Fig. 6b) and outstanding cycle performance for wide temperatures (0 and 50 °C). Similarly, we have previously proposed a new strategy to promote nitrate dissolution in ester electrolytes via crystalline H_2_O. As a typical case, the solubility of In(NO_3_)_3_-6H_2_O in commercial ester electrolyte is 0.2 M, which improves the cycling stability of LMB [91]. The additive can promote the generation of stable SEI with the inorganic-rich components on the LMA surface, inhibiting the formation of lithium dendrites, which is further demonstrated by the XPS results. After 300 cycles, the Li||NCM811 cell with In(NO_3_)_3_ additive has a capacity retention of 90.0% and an average CE of 99.3% (Fig. 6c), while the blank one only delivers capacity retention of 57.7%, with a low CE of 95.6%.

In addition to nitrate ions, there are other ions beneficial for the film formation. Zheng et al. designed an electrolyte composed of LiFSI in DME and 1,1,2,2-tetrafluoroethyl 2,2,3,3-tetrafluoropropyl ether (TTE) (3: 2 by molar) with 0.1 M LiPF_6_ (L-LDT) [92]. Based on Ab-initio molecular dynamics (AIMD) simulations along with the corresponding radial distribution functions (RDFs) analysis, a high density of Li-F peaks was observed at 1.92 Å (Fig. 6d). This provides direct evidence of PF_6_^−^ involvement in the main solvation sheath. Meanwhile, a stronger Li-O_DME_ peak indicates that the interaction between DME and Li^+^ is further strengthened. It is reasonable to infer that the enhanced interaction of Li^+^-DME and the reduced number of free DME molecules will improve the high-voltage (4.3 V) stability of the L-LDT electrolyte.

Hee-Tak Kim has proposed a novel sulfur-containing species, thiocyanate (SCN^−^), which exhibits both electron-donating and accepting properties for application in Li–S batteries [93]. The electron-deficient carbon atom provides high acceptor number (AN) functionality, while the electron-rich nitrogen and sulfur atoms provide high DN functionality. Molecular dynamics simulations and radial distribution functions show that high DN of SCN^−^ are more prone to participate in Li^+^ solvation sheath, facilitating the formation of a Li_3_N-rich AEI, thereby enhancing the reversibility of lithium metal anodes. Additionally, direct interaction between the high AN of SCN^−^ and polysulfides (PS) results in significantly high solubility of LiPS in LiSCN-based electrolytes, reinforcing the three-dimensional morphology of Li_2_S by the solution-mediator. Li–S batteries employing this anion chemistry approach exhibit outstanding lithium metal stability.

Li et al. proposed that the electrostatic interaction between anions and solvents in the electrolytes (1 M LiDFOB in the mixture of 2,2,2-trifluoro-N, N-dimethylacetamide (FDMA) and 1,1,2,3,3,3-hexafluoropropyl-2,2,2-trifluoroethylether (HTE) (1: 1 by volume)) has an important impact on the solvation structures [20]. Based on the results of molecular dynamics calculations and characterization tests, it was found that interactions of appropriate strength between anion (DFOB^−^) and solvent (FDMA) can promote more DFOB^−^ to enter the solvation sheath, fostering the development of a stable SEI and exhibiting high Li^+^ transport kinetics. Furthermore, benefiting from the fast ion transport kinetics through CEI film, excellent electrochemical performances for Li||LCO cell are achieved under the conditions of a high charge voltage of 4.5 V (Fig. 6e), the lean electrolyte of 15 μL (Fig. 6f), and a wide temperature range from − 20 to 60 °C.

LiF-rich SEI is usually a good strategy to improve interface issues. However, these fluorinated compounds (mainly LiPF_6_) can lead to serious side effects (e.g., the production of the toxic gas HF) at high temperatures, causing irreversible damage to the battery. Li et al. successfully obtained a LMB with a stable LiF-free SEI by using a fluorine-free electrolyte (1 M LiBOB + 0.5 M LiNO_3_ in DME) [94]. Specifically, when NO_3_^–^ is introduced into the electrolyte, it preferentially enters the inner solvated sheath layer and participates in the decomposition to generate SEI, and the interface is characterized as a composite interface mainly rich in B/O/N, with excellent stability and fast lithium-ion transport capacity. As a result, the Li||LFP battery can achieve a very-fast-charging capability at 100C and 60 °C.

In addition to the high temperature, Kong et al. proposed weak–strong anions (FSI^–^, NO_3_^–^) strategy to modulate the solvation sheath of lithium ions for achieving low-temperature LMBs [95]. The electrolyte based on weak FSI^–^ and the strong NO_3_^–^ anion properly balances ion transport and the stability of aluminum surfaces, making NCM622||Li batteries can normally work for 120 cycles at − 20 °C with capacity retention of 80%.

### Anion-Regulated Desolvation Process

As mentioned above, anions can regulate the solvation structure of Li-ion. Therefore, anions can regulate the desolvation process of Li-ion at the interface, which is very important for the charge transfer process. The coordinated solvents (anions), hydrogen bonds, and van der Waals forces can affect the desolvation process of Li-ion [80, 96–98]. Thus, the desolvation process can be understood as a decomposition coordination process with certain energy barriers [99]. That means the Li-ion will lose the coordinated solvents and anions through reorganization of the solvation structure. The process involves charge transfer, which is critical to the dynamics of the battery.

**Fig. 7:**
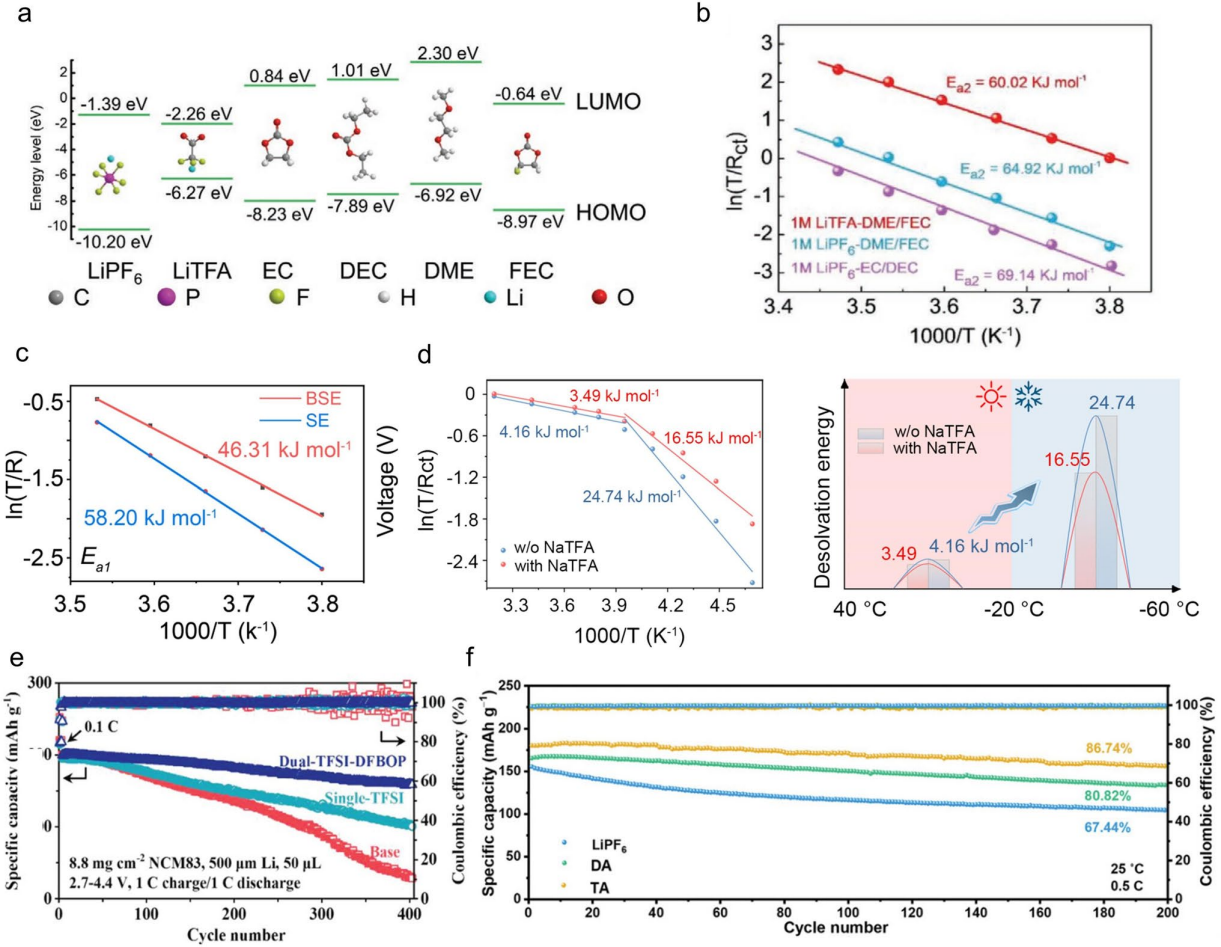
**a** Molecular orbital energy of different anions and solvents. **b** Activation energies derived from Nyquist plots [36]. Copyright 2020, Wiley‐VCH Verlag. **c** Activation energies of Li^+^ desolvation (E_a1_) in SE and BSE electrolyte [100]. Copyright 2023, Wiley–VCH GmbH. **d** Desolvation energy of Na^+^ derived from Nyquist plots and Schematic of desolvation energy in a wide temperature range [101]. Copyright 2024, National Academy of Sciences. **e** Electrochemical performance of Li||NMC83 coin cells in different electrolytes[102]. Copyright 2023, Wiley–VCH GmbH. **f** Cycling performance of Li||NCM811 cells using LiPF_6_, DA, and TA electrolytes at 0.5C and 25 °C [103]. Copyright 2024, Wiley–VCH GmbH

Anion regulation is a good optimization method to mediate the desolvation process of Li-ion. Li et al. used TFA^–^ to adjust the solvation sheath environment of Li^+^ and promote fast desolvation dynamics due to its high DN (34.0 kcal mol^–1^) [36]. By comparing the LUMO of various substances (Fig. 7a), it is evident that TFA^–^ is more prone to decomposition, preferentially forming a stable SEI rich in LiF and Li_2_O. E_a2_ represents the energy barrier of Li^+^ desolvation from the Li^+^ solvation sheath. Compared with the E_a2_ in LiPF_6_-EC/ DEC (69.14 kJ mol^–1^) and LiPF_6_-DME/FEC (64.92 kJ mol^–1^), the energy of LiTFA-DME/FEC (60.02 kJ mol^–1^) showed a slight decrease (Fig. 7b). The decreased energy barrier proves that TFA^−^ in the Li^+^ solvation sheath has a significant effect on the Li^+^ transport kinetics of SEI. Li et al. designed a dual-salt (0.5 M LiTFA, 0.5 M LiTFSI)-based electrolyte with G4 (tetraethylene glycol dimethyl ether) as the solvent in Li − O_2_ batteries [100]. This electrolyte slows down the decomposition of G4 and induces a SEI rich in inorganic substances. Electrostatic potential (ESP) calculations showed that the smaller volume of TFA^–^ is more concentrated in negative charge compared to TFSI^–^. As a result, Li^+^ is more readily attracted to the TFA^–^, thus reducing the binding strength to the G4. Further studies calculated the binding energies of G4 molecules to Li^+^ within solvated configurations containing different anionic coordination. As the number of TFA^–^ rises, the G4 binding energy decreases, suggesting that the enhanced anionic coordination induces a decrease in the Li^+^-solvent binding strength. The mean square displacement (MSD) suggests that the Li^+^ mobility is enhanced due to the weakened binding by G4 molecules. Meanwhile, compared with 1.0 M LiTFSI in G4, the TFA^–^ helps to reduce the energy barrier for desolvation (Fig. 7c). They also introduced TFA^–^ into G2-based (Bis(2-methoxyethyl)ether) electrolytes, enabling the regulation of solvation chemistry under low-temperature conditions to achieve highly stable SIB_s_ [101]. Compared with 1.0 M NaPF_6_ in G2, TFA^–^ anions gradually occupy the majority of the solvated structure, while the G2 molecules are squeezed out. The unique solvation structure accelerates the desolvation of Na^+^ by decreasing the desolvation energy from 4.16 to 3.49 kJ mol^−1^ and 24.74 to 16.55 kJ mol^−1^ at 40 to − 20 °C, respectively, compared with that in 1.0 M NaPF_6_-G2 (Fig. 7d).

Xu et al. developed a dual-anion modulator strategy for designing a good electrolyte for LMBs [102]. The TFSI^−^ anion can regulate the solvation structure of Li^+^ to produce a low Li^+^ desolvation energy, while DFBOP^–^ promotes the formation of high ion conductivity and sustainable inorganic-rich SEI on both sides. Impressive cycling performance of the cell was achieved in a dual-TFSI-DFBOP electrolyte, with high capacity retention of 80.5% after 400 cycles and a high CE of 99.9%. In contrast, the Li||LiNi_0.83_Co_0.11_Mn_0.06_O_2_ batteries showed a fast capacity fading, with only 14.4% and 51.5% capacity retention in base (1 M LiPF_6_ in FEC/EMC) and single-TFSI electrolyte, respectively (Fig. 7e). Moreover, Cheng et al. designed a ternary salts-based electrolyte (LiNO_3_, LiPF_6_, and LiTFSI in THF-FEC solvent), which exhibits reduced Li^+^ desolvation energy, making rapid charge transfer kinetics and a cold-resistant electrolyte [103]. Specifically, the strong interaction between NO_3_^–^ and Li^+^ weakens the interaction between PF_6_^–^/TFSI^–^ anions and Li^+^. In order to further investigate the binding energy of Li^+^ with one or more anions, DFT calculations were performed. It was found that Li^+^ had the lowest binding energy (− 4.62 eV) with the ternary anions. The optimized electrolyte demonstrates a high ionic conductivity of 3.39 mS cm^−1^ at − 60 °C and good compatibility with the lithium metal anode. Additionally, the lithium metal anode is effectively shielded from dendrite growth by a sturdy AEI derived from the anions. Moreover, the NCM811 cathode experiences reduced particle cracking due to a robust CEI. As shown in Fig. 7f, the ternary salts electrolyte enables Li||NCM811 cell to cycle stably at room temperature (25 °C) with a specific discharge capacity of 180.3 mAh g^−1^ and capacity retention of 86.74% after 200 cycles at 0.5C.

### Antioxidant Properties Affected by the Anion-Modulated Solvation

**Fig. 8:**
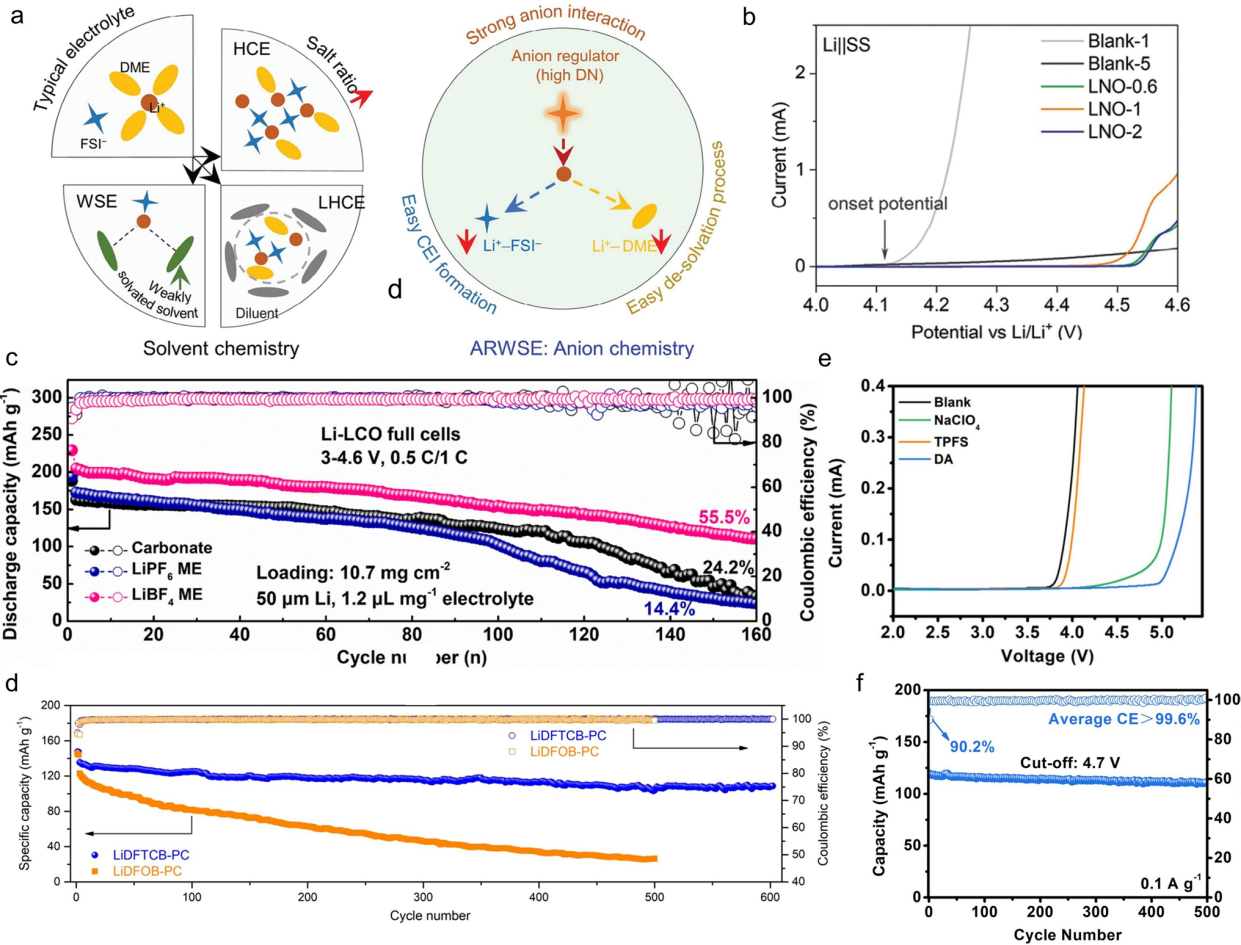
**a** Comparison between typical solvent chemistry and anion chemistry. **b** LSV profiles of Li||SS cells at a scan speed of 0.1 mV s^–1^ in different electrolytes[35]. Copyright 2023, Wiley–VCH GmbH. **c** Cycling performance of Li||LCO full cells with various electrolytes [109]. Copyright 2023, Wiley–VCH GmbH. **d** Cycling performance of the LCO||graphite full cells using LiDFTCB-PC and LiDFOB-PC electrolyte at room temperature [111]. Copyright 2023, Wiley–VCH GmbH. **e** LSV test of various electrolytes at a scanning rate of 2 mV s^–1^. **f** Cycling performance of Na||NVPF in 1 M NaPF_6_ in a mixed solvent of EC and DEC (1:1 by volume ratio) with trimethoxy(pentafluorophenyl)silane (TPFS) and NaClO_4_ [112]. Copyright 2022, Wiley–VCH GmbH

The developing of high-voltage batteries has faced challenges such as designing a suitable electrolyte, especially for the LMBs. Ether electrolytes are considered suitable for LMBs due to their compatibility with lithium metal. However, they cannot endure high voltages. Researchers usually apply high-concentrated electrolytes (HCE) [104, 105] or localized high-concentrated electrolytes (LHCE) [11, 106] strategies to improve the compatibility of both sides. Currently, the weakly solvating electrolytes (WSE) [107, 108] are confirmed an effective strategy to modify the electrochemical performance. The HCE strategy increases lithium salt concentration to reduce free solvents (increase the ratio of salt/solvent, Fig. 8a), constructing an anion-rich solvation structure which helps form a stable SEI. However, the high viscosity, low ionic conductivity, and high cost of HCEs limit their practical use. LHCE strategy mitigates these issues by using inert diluents (weak solvent), which can also maintain the anion-rich solvation structure. WSE strategy applies low-solvating-power solvents to encourage ion pairing, enhancing SEI stability but potentially reducing ionic conductivity. It should be noted that the above-mentioned strategies can be achieved by regulating the solvent (solvent chemistry). In comparison, anions can play a prominent role in electrolyte design (anion chemistry, Fig. 8a). By optimizing the structure and properties of anions, the solvation structure can be directly controlled. Unlike traditional methods, the anion chemistry is a new strategy to regulate the solvation structure, enabling the electrolyte good electrochemical performance like high-voltage stability.

Our group proposed a new strategy to tune the solvation structure of Li-ion by anion chemistry [35]. We utilized the high DN of NO_3_^–^ to coordinate with Li⁺, competing with DME in the solvation process. The NO_3_^–^ weakens the solvating ability of DME, and this competition reduces the number of solvated molecules, thereby lowering the likelihood of solvent oxidation at high potentials. As a result, an anion-regulated weakly solvating electrolyte (ARWSE) is formed (Fig. 8a). Even at a high voltage of 4.5 V, the electrolytes with NO_3_^–^ can normally work (Fig. 8b). Additionally, characterization experiments have confirmed that ARWSE promotes the formation of inorganic-rich components in the AEI/CEI, effectively suppressing continuous electrolyte decomposition and aluminum corrosion, thereby improving the antioxidant performance and coulombic efficiency of the battery. Li et al. achieved stable LMBs with DME electrolyte at 4.6 V (Fig. 8c), regulated by BF_4_^–^ anion [109]. The strong electrostatic attraction between Li^+^ and BF_4_^–^ causes more anions to be distributed in the first solvation shell, preferring to generate inorganic-rich SEI/CEI. Benefiting from its unique solvation structure, the oxidation resistance of the electrolyte is greatly increased (from 4.3 to 4.6 V). Guo et al. designed a THF-based electrolyte using dual salts of LiTFSI and LiNO_3_ [110]. They confirmed the interaction between the anion and the solvent, finding that the TFSI^–^ anion further enhanced the interaction between NO_3_^–^ and THF, and the reduction in the number of free THF molecules favored the enhancement of the oxidative stability of the electrolyte. The SiO_*x*_||NCM811 full cell using the designed ether-based electrolyte can still maintain 81.7% of the initial capacity after 500 cycles at 4.3 V, which is higher than that in the EC-based electrolyte (34.7%).

Cui and colleagues successfully synthesized a cyanide-containing lithium salt: lithium difluoro(1,2-dihydroxyethane-1,1,2,2-tetracarbonitrile) borate (LiDFTCB) [111]. They found that using LiDFTCB/PC electrolyte, the graphite||LCO full cell demonstrates high capacity retention of 80.2% after 600 cycles at 25 °C and 0.5C. In sharp contrast, LiDFOB-PC shows poor electrochemical compatibility with the graphite||LCO cell, delivering a dramatically low-capacity retention of 21.5% (Fig. 8d). The HOMO energy level of DFTCB^–^ is lower than that of DFOB^–^, indicating higher oxidative stability of DFTCB^–^. Additionally, electrochemical characterization revealed that the oxidation stability of the LiDFTCB-based electrolyte is superior to that of the LiDFOB-based electrolyte, ascribing to the replacement of the − C = O moieties by four − C≡N groups. Results reveal that more DFTCB^–^ anions and less PC solvent are involved in the solvation structure of Li^+^. The high coordination number of DFTCB^–^ with Li^+^ will lower the LUMO energy level of the anion, which favors the formation of inorganic-rich SEIs on the anode. This prevents severe capacity degradation of the battery at 50 °C. In comparison, LiDFOB undergoes severe decomposition at 50 °C. While using LiDFTCB salt, no CO_2_ gas was detected at 50 °C.

In addition to the application of anion-regulation for lithium batteries, Ma et al. stabilized a electrolyte (1 M NaPF_6_ in EC/DEC) for 4.7 V SIBs by rationally configuring the solvated structure of the electrolyte with NaClO_4_ and trimethoxy(pentafluorophenyl)silane (TPFS) as dual additives (Fig. 8e, f) [112]. Here ClO_4_^−^ as the voltage-stimulated response species tends to rapidly move to the cathode surface during the charging process, then binds with Na^+^ and solvents to form polymer-like structures (ClO_4_^−^-Na^+^-solvent), which dramatically reduces the continuous solvent decomposition at high voltages. Meanwhile, the Si − O derived from TPFS can effectively capture adverse species such as HF and H_2_O, generating Si − F and Si − O, which could well protect CEI integrality and suppress CEI dissolution. Thus, these two additives increase the oxidative stability of the carbonate electrolyte from 3.77 to 4.75 V. Na||Na_3_V_2_(PO_4_)_2_O_2_F cells using the optimized electrolyte achieve a stable cycling performance at 4.7 V, with 93% capacity retention after 500 cycles and 99.6% average CE.

## Flame-Retardant Applications by Halogen Anions

In addition to the requirement of high energy density for a battery, the safety issue is also very important. Halogen-containing flame retardants can be divided into fluoride, chloride, and bromide-containing flame retardants. When subjected to heat, the halogen can produce free radicals, inhibiting the combustion process [113]. At present, F-, Cl-, and Br-type flame-retardant additives are widely used, while I-type flame-retardant additives are less used. This is because the C-I bond is too weak to remain stable [114]. Among the halogen-containing flame retardants for LIBs, F-substituted compounds are the most studied. Fluorinated compounds can increase the flash point of the electrolyte, reduce the flammability of the electrolyte, and promote the generation of SEI film on the anode surface to improve the performance of the battery [115–118].

**Fig. 9:**
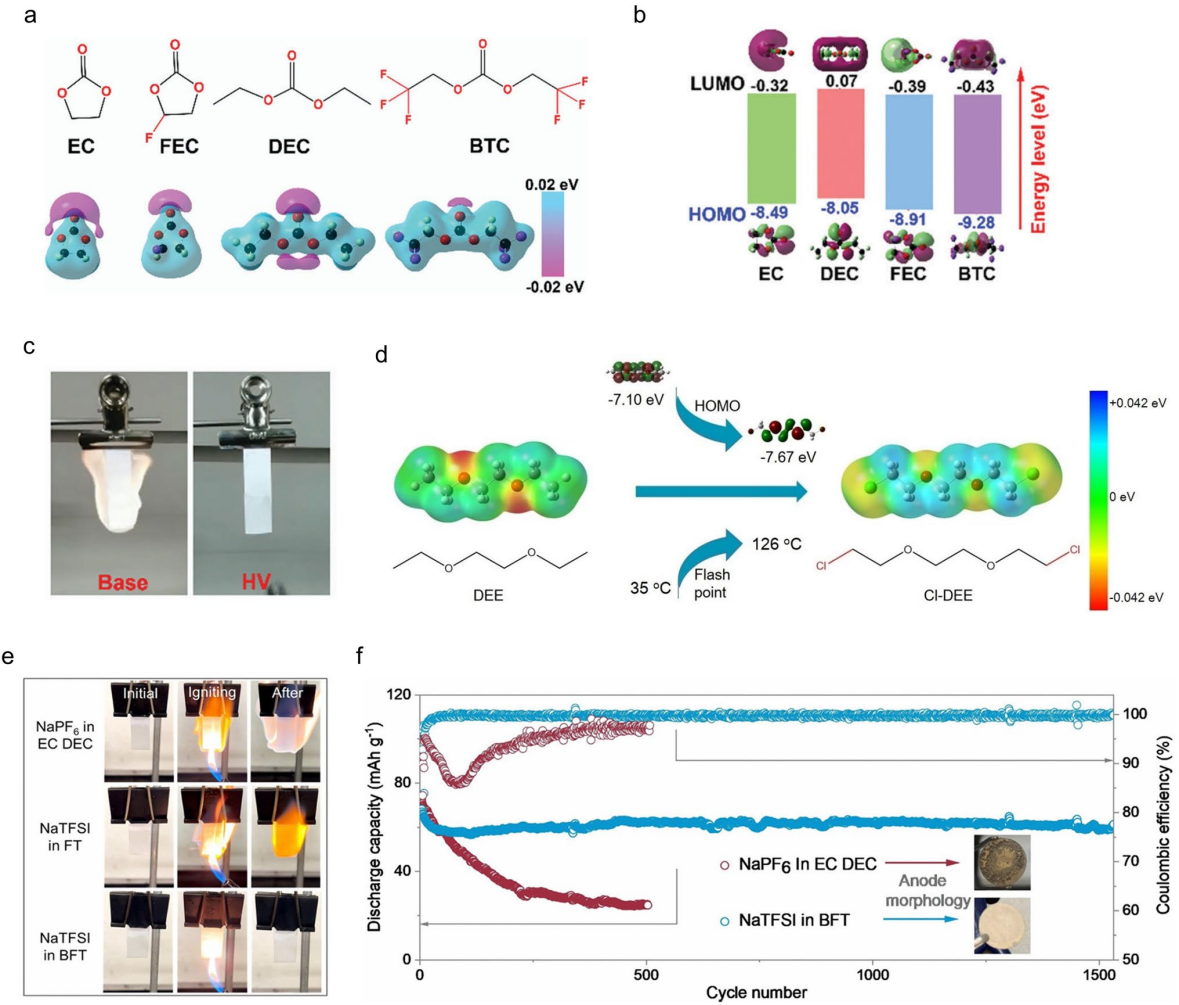
**a** Molecular structure of various solvents and their corresponding charge distributions. **b** Molecular orbital energies of different solvents. **c** Flammability tests of the HV electrolyte composed of 1 M LiPF_6_ in a mixture of FEC and bis(2,2,2-trifluoroethyl) carbonate (BTC) and base electrolyte:1 M LiPF_6_ in a mixture of EC and DEC [119]. Copyright 2022, The Royal Society of Chemistry. **d** Design scheme and molecular structures of DEE and Cl-DEE [120]. Copyright 2022, Wiley–VCH GmbH. **e** Ignition experiments of NaPF_6_ in EC/DEC, NaTFSI in FT and NaTFSI in BFT electrolytes. **f** Cycling performance of Na_3_V_2_(PO_4_)_3_||Na cells in different electrolytes at 300 mA g^−1^ [124]. Copyright 2024, Royal Society of Chemistry

The introduction of fluorine atoms into the ester solvents results in a lower LUMO (Fig. 9a, b), which can be preferentially reduced on the lithium metal surface, resulting in the formation of a LiF-rich solid electrolyte interface, which inhibits the growth of lithium dendrites [119]. Therefore, the optimized electrolyte (1 M LiPF_6_ in FEC BTC) used in Li||NCM811 batteries can operate within a wide operating temperature range of − 30 to 70 °C. More importantly, during flame-retardancy tests, the optimized electrolyte is non-flammable (Fig. 9c), implying its stability at high temperatures and safety in practical use. Similarly, researchers also introduced fluorine atoms into ether solvents and found that fluorine atoms not only improve the flame-retardant properties of the solvent but also enhance the oxidation stability of the electrolytes [120]. Compared with the C-F bond (115 kcal mol^−1^), the C–Cl bond (83.7 kcal mol^−1^) is more prone to break, with the production of chloride radicals, which can effectively capture the highly active H· and generate HCl. The generated HCl can also capture ·OH and generate Cl· which can re-participate in the above reaction to effectively suppress the progress of combustion. As shown in Fig. 9d, the Cl-DEE (1,2-bis(2-chloroethoxy)-ethyl ether) has a lowered HOMO than the DEE, indicating improved oxidation stability. Moreover, the flash point of Cl-DEE is 126 °C, which is much higher than the 35 °C of DEE. The electrolyte (LiFSI in Cl-DEE/TTE, 1:1.6:3 by molar) provides excellent electrochemical stability even under ultra-high-voltage conditions (4.6 V).

Solid electrolytes offer a higher level of safety compared to liquid electrolytes [121–123]. Huang et al. designed a novel solid-state polymer electrolytes (SPEs) based on nonflammable polyurethane, which is covalently bound with reactive brominated flame retardant on the backbone [39]. Brominated flame retardants can participate in the formation of LiBr-rich SEI, which can enhance the stability of the electrolytes. Furthermore, the prepared polymer electrolytes have good electrochemical properties. The Li||SPEs||Li symmetric battery can undergo stable cycling for more than 2100 h. The Li||SPEs||LiNi_0.6_Co_0.2_Mn_0.2_O_2_ battery maintains capacity retention of 85.2% after 330 cycles at 0.3C. In contrast, the cell with unimproved electrolytes showed a reversible specific capacity of 162 mAh g^−1^ during the activation cycles and then decayed to 68 mAh g^−1^ after 200 cycles.

There is a wide variety of brominated compounds with excellent flame-retardant effects and wide applications, but relatively few studies have been conducted in LIBs. Researchers have developed a novel brominated flame-retardant 2-bromo-1-(2-bromoethoxy) ethane (BBE) solvent to improve the flame-retardant efficiency of SIBs [124]. Moreover, due to the low energy barrier for the dissociation of free radical scavengers, the Br-based flame retardants show the best response compared with the F-based, Cl-based, and P-based flame retardants (Fig. 9e). Br-based solvents lead to the formation of NaBr-rich SEI on the sodium metal. Due to its high ion conductivity, it inhibits the growth of sodium dendrites. Additionally, the Na||Na_3_V_2_(PO_4_)_3_ battery exhibits excellent cycling performance with a high capacity retention of 91.3% after 1500 cycles (Fig. 9f). In contrast, the capacity retention of cells utilizing NaPF_6_ in EC/DEC decreased to 80% after only 60 cycles.

## Other Effects of Anions

**Fig. 10:**
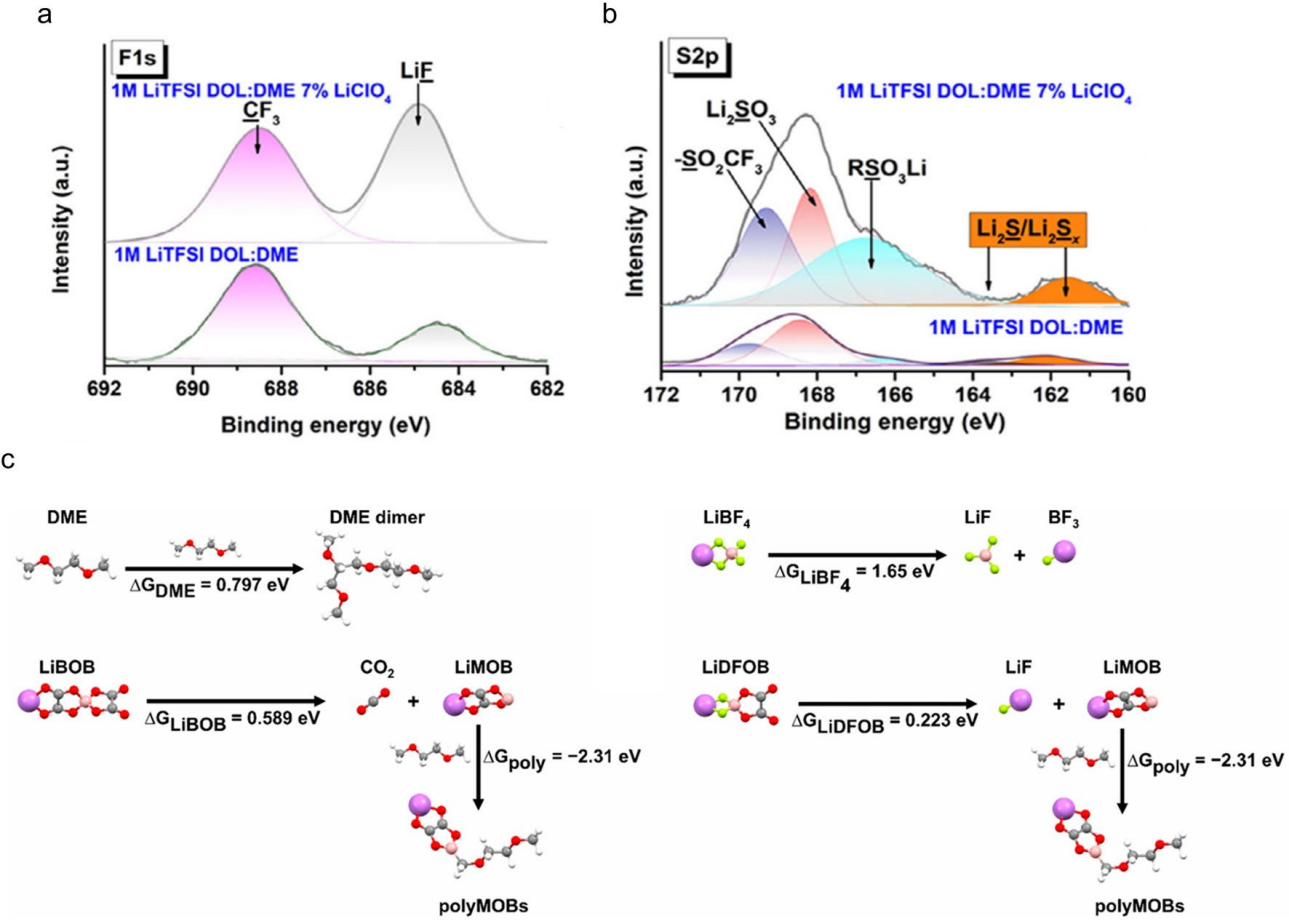
XPS spectra of the formed SEI in different electrolytes for **a** F 1*s* and **b** S 2*p* before and after the adding of LiClO_4_ [125]. Copyright 2019, Published by Elsevier. **c** Optimized geometries and the corresponding decomposition reaction free energies of DME, LiBF_4_, LiBOB, and LiDFOB at high-voltage [126]. Copyright 2023, American Chemical Society

In addition to what we mentioned above, there are also other effects influenced by anions. Huang et al. reported that ClO_4_^–^ can change the decomposition path of TFSI^–^ as catalysis, enabling the complete decomposition of TFSI^–^, thereby forming an excellent SEI [125]. Specifically, high-resolution spectra of F 1*s* (Fig. 10a) clearly showed higher intensity of LiF in SEI after adding LiClO_4_, further unambiguously confirming that the association generated by LiClO_4_ contributes to the decomposition reaction of LiTFSI. A similar phenomenon was also found for high-resolution spectra of S 2*p* (Fig. 10b). The relative content of S atoms was at a low level without adding LiClO_4_, represented by the weak peak intensity of the reduction products. With increasing the content of LiClO_4_, the content of RSO_3_Li, Li_2_S, Li_2_S_*x*_, and Li_2_SO_3_ increased significantly. As a result, the use of LiClO_4_ effectively improves the CE and cycle life of the LMBs.

Xie et al. observed that LiDFOB can change the traditional decomposition path of ether solvents, thereby improving the high-voltage stability of the electrolytes (Fig. 10c) [126]. It is found from the calculation that LiDFOB can preferentially catalyze DME to generate LiF and lithium mono(oxalate)borate (LiMOB). LiMOB can further trigger the polymerization reaction of DME to generate polyMOBs, thereby inhibiting the continuous decomposition of DME at the positive electrode. A thin and uniform organic–inorganic composite CEI was formed on the surface of LCO with the LiDFOB salt. This unique structure can effectively protect the LCO cathode and inhibit adverse reactions at the positive electrode. In addition, new chemical bonds and functional groups can be identified by Fourier transform infrared spectroscopy (FTIR) after the electrolyte decomposition, further supporting the pathway switching mechanism that altered the degradation process of the DME. Zhao et al. also reported that a small amount of LiDFOB additive can alter the decomposition pathway of trans-difluoroethylene carbonate (DFEC) [127]. The presence of LiDFOB can significantly induce the direct defluorination of DFEC, accompanied by the formation of inorganic species such as LiF and Li_*x*_BF_*y*_. The defluorinated DFEC with high reactivity can polymerize into highly elastic poly (VC), which will interweave with the concurrently generated LiF, forming a distinctive SEI with a LiF-dominated inner layer and a LiF-polymeric interwoven outer layer. This SEI film exhibits excellent mechanical stability and good ion transport properties, significantly improving the cycling and rate performance of the silicon anode.

Anion chemistry is also widely used in dual-ion battery [128, 129]. For example, Cui et al. designed an anion-permselective polymer electrolyte that preferentially coordinates PF_6_^–^ with a quaternary ammonium group, enhancing PF_6_^–^ desolvation and improving the structural integrity of graphite electrodes while increasing electrolyte oxidative stability [130]. Tang et al. developed a high-concentration LiFSI electrolyte, which promotes the formation of a stable SEI layer, enhancing the full-cell energy density by suppressing gas formation at high voltages [131].

## Summary Outlook

We have emphasized the roles of different anions in the electrolyte and summarized the effects of anions on surface interfacial chemistry, solvation structure, and electrochemical stability. Although the anion chemistry in electrolytes is still in its early stages of research, it has made significant progress in energy storage, safety issues, interfacial chemistry, and kinetics. This research direction is increasingly gaining attention and will undoubtedly guide research efforts in the field of energy storage. For specific battery systems, significant stability improvements can be achieved by selecting suitable anion(s). Here we give a summary of the characteristics of the mentioned anions, which is also shown in Table 1. It should be noted that DN is selected as a representative parameter to describe the coordination ability of the anions and solvents with Li-ions, helping to infer the solvation structure which directly connected with SEI formation. However, parameters such as LUMO, HOMO, activation energy, and binding energy also play a role in selecting anions and solvents. A more accurate choice may be made with the help of DFT calculations.

**Table 1:** The role of different anions and their limitations. (The structures of these anions are shown in Fig. 1) Here n/a means not applicable

Anion	Primary Roles	Limitations
FSI⁻	Stable SEI; wide temperature range stability; high ion transport; SEI repair agent	Aluminum corrosion at high voltages
TFSI⁻	Hydrolysis resistance; stable SEI; high ionic conductivity; thermal stability	Aluminum corrosion at high voltages
NO_3_^–^	N-rich SEI; solvation structure regulator; adsorbent	Low solubility in ester solvents; sacrificial agent
PF_6_^–^	Stable CEI; aluminum current collector protection	Poor hydrolytic and thermal stability; sensitive to moisture
DFOB⁻	Stable CEI; alters solvent decomposition pathways; high voltage stability	Low solubility; produces harmful gases at high temperatures
CIO_4_^–^	Stable CEI; synergistic film formation	Strong oxidizing properties; risk of side reactions at high voltages
FFF⁻ & FBF⁻	Stable SEI (LiF/LiBr-based SEI); good cathodic degradability; high Li^+^ selectivity	n/a
FEA⁻	SEI repair agent; dissolves organic impurities; high molecular dipole; high DN; high *t*_Li+_	Excessive SEI dissolution; nucleophilic reactivity
ETFSI⁻	SEI repair agent; dissolves organic impurities	n/a
SCN⁻	Stable SEI; high oxidation resistance	Poor conductivity
CN⁻	Synergistic film formation; removes water and acid	Strong oxidizing properties
TFA⁻	Solvation sheath regulator; rapid ion transport; enhances oxidation resistance	Lower dissociation degree; lower overall conductivity
DFBOP⁻	Inorganic-rich SEI; high ionic conductivity	n/a
BF_4_^–^	Good oxidative stability; robust SEI; high temperature stability; corrosion resistance	Limited ionic conductivity; low solubility
I^–^/I_3_^–^	Repairs agent of dead lithium; improves fast-charging stability in lithium metal batteries	n/a
F-, Cl-, Br-, I-based flame retardants	Flame retardancy; stable SEI; enhances high-temperature safety	Iodine-based compounds are less stable

The FSI⁻ anion and the TFSI⁻ anion play crucial roles in protecting the negative electrode, forming a stable SEI, and enhancing ion transport performance. The FSI⁻ anion exhibits good electrochemical stability at low temperatures, making it suitable for wide temperature range applications. While the TFSI⁻ exhibits resistance to hydrolysis and possesses high ion conductivity and commendable thermal stability. However, electrolytes based on FSI⁻ and TFSI⁻ cannot effectively passivate aluminum foil current collectors, especially at high operating voltages.

Nitrate anions in batteries have several advantages such as forming a protective film on the electrode surface. They possess good adsorption properties, helping to regulate the chemical properties of the electrode interface. They can also adjust the solvation structure of solvent molecules in the electrolyte, optimizing ion transport performance. NO_3_⁻ complex with Al^3+^ on the surface of aluminum foil forms a passive layer containing AlN, inhibiting the corrosion of aluminum. Additionally, nitrate anions are inexpensive, and price-friendly. However, nitrate anions have low solubility in ester solvents, limiting their application in certain electrolyte systems. Furthermore, as sacrificial additives, nitrate anions are gradually consumed during battery reactions and need periodic replenishment, which affects the long-term usage of the battery.

PF_6_⁻ anion can form a stable protective layer on an aluminum current collector, effectively preventing aluminum corrosion and significantly extending battery lifespan. Additionally, during battery operation, PF_6_⁻ anion decomposes to form a conductive SEI enriched with LiF. This interface layer not only enhances the stability of the cathode but also improves battery performance. However, shortcomings such as poor hydrolytic stability, insufficient thermal stability, and sensitivity to moisture limit their application in more demanding or extreme environments.

LiDFOB forms a new structure within the IHP, facilitating the formation of a robust CEI, thereby preventing the dissolution of the active material. This interface layer effectively prevents adverse reactions between electrolyte components and solvents, significantly extending battery life and enhancing electrolyte stability. Additionally, LiDFOB can alter the decomposition pathway of some solvents, enhancing battery stability at high voltages and further improving battery performance.

The role of ClO_4_⁻ in batteries enhances performance by optimizing the formation and stability of the CEI and promoting Li^+^ transport. Moreover, it has the role of a catalyst when coupled with some anions/solvents, making a good SEI formation to prevent lithium dendrite growth and extend battery life. However, electrolytes containing ClO_4_⁻ have strong oxidizing properties, which can easily trigger side reactions at higher voltages, causing battery swelling and posing safety issues.

LiFFF and LiFBF are more easily electrochemically reduced on the anode surface and form LiF/LiBr-rich SEIs to passivate the lithium metal anode. LiFEA dissolves organic impurities and promotes the enrichment of inorganic substances, optimizing and stabilizing the SEI layer. In addition, another salt, LiETFSI, is superior to LiFEA in dissolving oxygenated organic compounds in the SEI while reducing the dissolution of inorganic components such as LiF. The SCN⁻, as an electrolyte additive in LMB, significantly enhances the performance and stability of lithium metal anodes by its solvation properties and the formation of a stable SEI. The CN⁻ in LMB primarily functions to inhibit the dissolution of Co ions. It achieves this by forming a dual-layer CEI consisting of an inner LiF layer and an outer layer rich in B − F/ − CN organic structures. These actions collectively enhance the stability and performance of the battery.

The high DN of TFA⁻ regulates the solvation sheath environment of Li^+^ and promotes rapid dissolution kinetics. The main role of DFBOP⁻ in sodium-ion batteries is to preferentially decomposition on both the positive electrode and metal negative electrode sides, forming stable and robust CEI/AEI. These interfaces contain inorganic substances, significantly enhancing the stability. The strong electrostatic attraction between Li^+^ and BF_4_⁻ leads to more anions distributed in the first solvated shell layer, which greatly reduces the number of free anions. Due to its unique solvated structure, the oxidation resistance of the cell is greatly enhanced.

The use of a reversible I⁻/I₃⁻ redox reaction can deal with the deposition of dead lithium on the anode, which is an effective strategy to improve the performance of LMB even under fast-charging conditions. Moreover, halogen-containing flame retardants play a critical role in enhancing the safety and performance of battery electrolytes. By capturing free radicals and forming stable SEI, these compounds mitigate the risks associated with high temperatures and dendrite formation, while also significantly improving the overall electrochemical stability of the batteries.

In summary, the anions’ involvement in the solvation structure can greatly influence the electrochemical stability of the electrolyte. However, characterization methods are not yet sufficient and mature enough to obtain accurate solvation structure and dynamic information, especially during battery cycling. It is also important to elucidate the formation and composition of the interfacial film generated by anions and solvents. The design of a uniform and stable SEI is crucial for enhancing the electrochemical stability of the electrolyte and stabilizing both negative and positive electrode materials. This requires a comprehensive understanding of the thermodynamics and kinetics of anion decomposition. Furthermore, studying the plating/stripping behavior of metal anodes (Li^0^, Na^0^) is crucial for improving the cycle stability of metal batteries. Analyzing the role of anions in these processes is an effective strategy to achieve the goal, sometimes the combination of the multiple anions is better. In the future, there will be further exploration of novel anion chemistry. Through the structural design of the anions, it will be possible to precisely control the anion chemistry to enhance energy density, cycle life, and safety of the batteries. At the same time, by integrating the popular artificial intelligence computing technologies, it is possible to better predict and optimize the performance of electrolytes, driving innovation and development in this field. In conclusion, the future development of the electrolyte field can be achieved not only through the design, development, and application of anions but also through the development of new solvents, laying a solid foundation for the next generation of high-performance and safe battery technologies.
